# Androgen deprivation induces neuroendocrine phenotypes in prostate cancer cells through CREB1/EZH2-mediated downregulation of REST

**DOI:** 10.1038/s41420-024-02031-1

**Published:** 2024-05-22

**Authors:** Dayong Zheng, Yan Zhang, Sukjin Yang, Ning Su, Michael Bakhoum, Guoliang Zhang, Samira Naderinezhad, Zhengmei Mao, Zheng Wang, Ting Zhou, Wenliang Li

**Affiliations:** 1 Texas Therapeutics Institute; Brown Foundation Institute of Molecular Medicine, University of Texas Health Science Center at Houston, Houston, TX USA; 2 University of Texas MD Anderson Cancer Center UTHealth Graduate School of Biomedical Sciences, Houston, TX USA; 3 Present Address: Department of Oncology, Shunde Hospital, Southern Medical University, Foshan, China; 4 Present Address: The First People’s Hospital of Shunde, Foshan, China; 5 Present Address: Department of Pain, Union Hospital, Tongji Medical College, Huazhong University of Science and Technology, Wuhan, China

**Keywords:** Urethral neoplasms, Gene silencing

## Abstract

Although effective initially, prolonged androgen deprivation therapy (ADT) promotes neuroendocrine differentiation (NED) and prostate cancer (PCa) progression. It is incompletely understood how ADT transcriptionally induces NE genes in PCa cells. CREB1 and REST are known to positively and negatively regulate neuronal gene expression in the brain, respectively. No direct link between these two master neuronal regulators has been elucidated in the NED of PCa. We show that REST mRNA is downregulated in NEPC cell and mouse models, as well as in patient samples. Phenotypically, REST overexpression increases ADT sensitivity, represses NE genes, inhibits colony formation in culture, and xenograft tumor growth of PCa cells. As expected, ADT downregulates REST in PCa cells in culture and in mouse xenografts. Interestingly, CREB1 signaling represses REST expression. In studying the largely unclear mechanism underlying transcriptional repression of REST by ADT, we found that REST is a direct target of EZH2 epigenetic repression. Finally, genetic rescue experiments demonstrated that ADT induces NED through EZH2’s repression of REST, which is enhanced by ADT-activated CREB1 signaling. In summary, our study has revealed a key pathway underlying NE gene upregulation by ADT, as well as established novel relationships between CREB1 and REST, and between EZH2 and REST, which may also have implications in other cancer types and in neurobiology.

## Introduction

Prostate cancer stands as the second most prevalent cancer and the second leading contributor to cancer-related mortality among men in the United States. The American Cancer Society estimates about 299,010 new cases and about 35,250 deaths from prostate cancer in the United States in 2024. Androgen deprivation therapies (ADT) focusing on the androgen receptor (AR) remain the cornerstone treatments for prostate cancer (PCa). Though ADT initially displays effectiveness, the majority of tumors inevitably experience recurrence and evolve into castration-resistant prostate cancer (CRPC). The process of lineage plasticity has gained prominence as a pivotal mechanism driving the progression of CRPC [1–3].

Approximately 20% of fatal CRPC cases exhibit a significant presence of neuroendocrine-like tumor cells, referred to as treatment-related neuroendocrine prostate cancer (t-NEPC or CRPC-NE, herein termed NEPC) [4–6]. These NEPC cells are thought to originate, at least partially, from neuroendocrine differentiation (NED) of adenocarcinoma cells [7, 8]. Key attributes of NEPC cells encompass the loss of androgen receptor signaling, resistance to ADT, and heightened expression of neuroendocrine markers, including neuron-specific enolase (ENO2), synaptophysin (SYP), tubulin, beta 3 class III (TUBB3), chromogranin A (CHGA), and chromogranin B (CHGB) [4, 9–11]. Nonetheless, the intricate mechanisms governing the induction of NE markers by ADT remain incompletely elucidated.

CREB1 and REST are two established master activator and repressor of neuronal genes in the brain, respectively [12–14]. In epithelial cancer cells, REST has been reported to act as a tumor suppressor [15]. REST represses NE gene expression in prostate cancer cells [16–19]. We and others have demonstrated that activation of CREB1 signaling is critical for ADT-induction of NE markers [20–23]. It is still unclear how CREB1 activation induces NE markers, and there has been no direct link established between CREB1 and REST in the context of ADT-induced NED.

Polycomb repressive complex 2 (PRC2) represses gene transcription by catalyzing methylations on histone H3 lysine 27 (H3K27me) [24–27], which are repressive histone marks. The major enzyme for catalyzing histone H3K27 methylations is EZH2 (Enhanced Zeste Homolog 2) [28–30]. EZH2 is overexpressed in several solid tumors, such as prostate, breast, and lung cancers [29, 31–38]. EZH2 expression and its PRC2 activity are particularly high in NEPC [39–41]. In prostate cancer, EZH2 is known to collaborate with AR in promoting the progression of AR-positive CRPCs through both H3K27me3-dependent and -independent mechanisms [42, 43]. On the other hand, how EZH2 promotes the progression of AR-negative NEPC remains an open question [41, 44].

REST and EZH2 are partners in transcriptional repression in mammalian cells [45–47]. REST has been shown to physically interact with EZH2, which can lead to the recruitment of PRC2 complex to repress REST targets, such as neuronal genes [48–50]. In addition to recruitment by REST, EZH2 has been shown to methylate REST, which stabilizes REST when bound to the RE1 sites of target genes [51].

Previously, we reported that CREB1 signaling acts through EZH2’s PRC2 activity to induce NE markers [20]. However, the critical link between ADT-CREB1-EZH2 pathway and NE induction is still missing. As an epigenetic repressor in this NED context, as we and others have shown [20, 40], EZH2 is expected to repress some transcription regulators that in turn suppress NE marker expression.

To better understand the mechanisms of NED in prostate cancer cells, here we investigated the links between ADT, CREB1, EZH2, and REST. We found that CREB1 activation leads to the downregulation of REST, and ADT induces NED through the CREB1/EZH2/REST pathways. Intriguingly, REST itself is an epigenetic target of EZH2 in NEPC cells. Our study has thus provided critical new information regarding how ADT, CREB1, and EZH2 induce NED, and also revealed a direct repression of REST by CREB1 signaling and by EZH2’s epigenetic regulation.

## Results

### REST is downregulated in NEPC

**Fig. 1:**
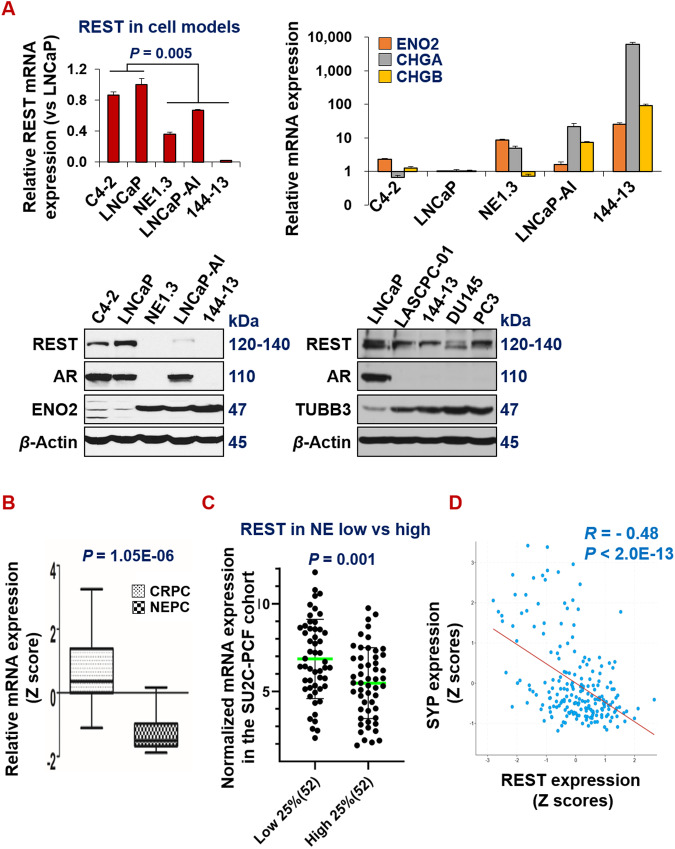
REST is downregulated in NEPC. **A** qPCR and Western blotting for expression patterns of the indicated genes and proteins in panels of human prostate cancer cell lines. LNCaP and C4-2 cells represent NE-/low cell models, while other cells represent NE + /high cell models. Y-axis shows relative fold changes in mRNA expression, normalized to GAPDH. Error bars in PCR results represent standard deviation (s.d). Beta actin was examined as the loading control in Western blotting. **B** REST mRNA is significantly lower in NEPC (CRPC-NE) than in adenocarcinoma CRPC (CRPC-adeno) (Beltran_NM2016) [39]. **C** REST mRNA in the top 25% NE-high samples versus the bottom 25% NE-low samples of CRPC in the SU2C-PCF dataset [52]. **D** Expression of NE marker SYP negatively correlates with that of REST in the SU2C-PCF dataset. REST correlations for additional NE markers (ENO2, CHGA, CHGB, and TUBB3) are presented in Supplementary Fig. S1.

To determine the expression patterns of REST in adenocarcinoma and neuroendocrine prostate cancer (NEPC), we analyzed its expression patterns in a panel of androgen-dependent prostate cancer and NEPC cell lines, and patient samples. In line with the literature, REST mRNA and protein levels are lower, while NE markers are higher, in NE+ PCa cell lines NE1.3, LNCaP-AI, LASCPC-01, and 144-13 than in NE- cell lines C4-2 and LNCaP (Fig. 1A). Similar expression patterns were found in prostate cancer patient samples. Upon examining the Beltran_NM2016 RNA-seq dataset [39], we found that REST is significantly lower, while NE markers are significantly higher, in 15 NEPC samples compared to 34 CRPC-adenocarcinoma samples (Fig. 1B, *P* = 1.05E-06). In another well-cited prostate cancer dataset (SU2C-PCF_PCa [52]), based on the NE scores provided in the dataset, we compared the cases in the NE score top 25% versus those in the NE score bottom 25%. As shown in Fig. 1C, REST mRNA expression is significantly lower in the top 25% of samples (*P* = 0.001). As expected, REST expression negatively correlates with NE markers in prostate cancer patient samples (Fig. 1D and Supplementary Fig. S1). Overall, these results confirm that REST expression is downregulated in NEPC.

### REST inhibits NE marker expression, ADT resistance, and tumor progression

**Fig. 2:**
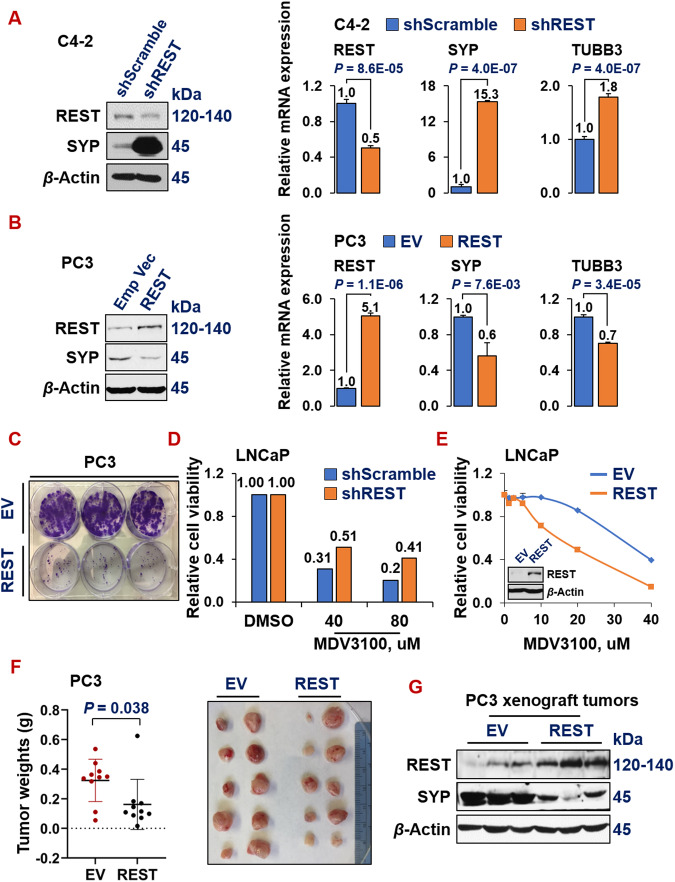
REST inhibits NE marker expression, ADT resistance and tumor progression. Western blotting and qPCR for REST and NE markers in NE-/low C4-2 cells upon REST silencing (**A**) and in NE + PC3 cells upon overexpressing REST cDNA (**B**). Y-axis shows relative fold changes in mRNA expression, normalized to beta actin. Error bars in PCR results represent standard deviation (s.d). Western blotting was carried on whole cell lysates from the indicated cell lines and for the indicated proteins. **C** Colony formation assay of PC3 cells carrying empty vector (EV) or REST cDNA. The two cell lines were seeded in triplicates in a 6-well plate, at 1000 cells/well and cultured for 14 days, followed by staining with crystal violet. **D** Viability and proliferation of LNCaP cells expressing either scramble shRNA or shREST, upon treatments of 40 µM or 80 µM of ADT drug MDV3100 for 3 days. **E** Cell viability and proliferation of LNCaP cells carrying either empty vector or REST cDNA, upon treating with a series doses of ADT drug MDV3100 for 3 days. Y-axis shows relative cell viability and proliferation, normalized to DMSO control. The experiment was carried out in quadruplicates each time, twice with similar results. Insert: Western blotting shows REST overexpression in PC3-REST cells. **F** Subcutaneous xenograft tumor growth of PC3 cells carrying either EV or REST cDNA in NOD/SCID male mice. Left: tumor weights at euthanization (*P* = 0.038) 8 weeks after implantation. Right: images of the xenograft tumors at euthanization (5 mice for each cell line, *n* = 10 tumors). **G** Western blotting for REST and NE marker SYP protein levels in xenograft tumors from PC3-EV and PC3-REST cells.

To investigate the role of REST in prostate cancer progression, we modulated REST expression via cDNA over-expression and shRNAs knock-down systems in prostate cancer cells, followed by examining NE marker expression and phenotypes of prostate cancer cells in vitro and in vivo. Silencing REST in C4-2 (NE-/AR+) adenocarcinoma cells leads to induction of NE markers (Fig. 2A). Conversely, overexpressing REST in PC3 cells (NE + /AR-) [53] downregulates NE markers (Fig. 2B). Phenotypically, REST overexpression dramatically reduced colony formation of PC3 cells (Fig. 2C). Moreover, REST silencing increased (Fig. 2D), while its overexpression decreased (Fig. 2E), the viability of LNCaP cells under treatment with ADT drug MDV3100 (Enzalutamide). To examine REST’s role in regulating prostate tumor growth in mouse xenografts, PC3-EV (empty vector) and PC3-REST cells were implanted subcutaneously (s.c.) in NOD/SCID male mice for 24 days. Xenograft tumors from PC3-REST cells are significantly smaller than tumors from PC3-EV cells (Fig. 2F). Additionally, the expression of NE marker SYP was reduced in xenograft tumor tissues exhibiting REST overexpression (Fig. 2G). These results demonstrated that REST is a tumor suppressor gene in prostate cancer.

### ADT downregulates REST both in vitro and in vivo

**Fig. 3:**
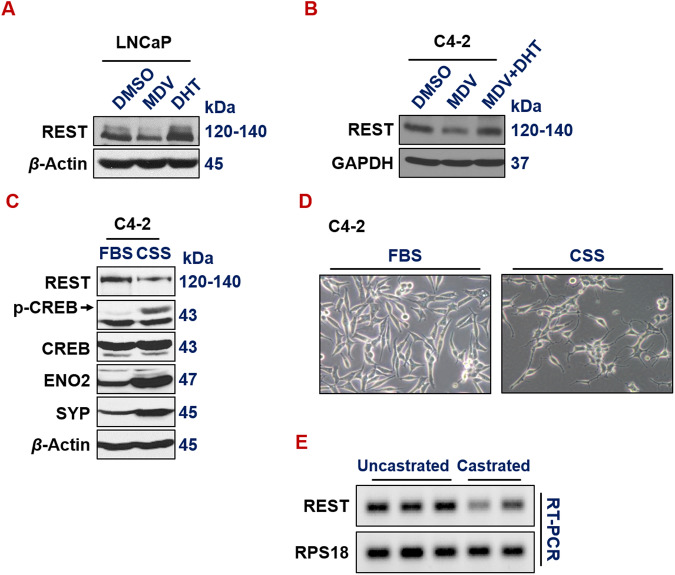
ADT downregulates REST in vitro and in vivo. **A**, **B** Western blotting of REST in LNCaP and C4-2 cells treated with ADT drug MDV3100 (10 µM, 72 h), androgen DHT (25 nM, 24 h) or both. **C** Western blotting of whole cell lysates from C4-2 cells growing in media with regular FBS or CSS (charcoal stripped serum) for 7 days. Culturing in CSS media that is deprived of hormones is another common ADT approach in vitro. **D** Morphology of C4-2 cells growing in media with regular FBS or CSS. **E** RT-PCR and DNA gel electrophoresis of REST and loading control RPS18 in LNCaP xenograft tumors growing in uncastrated or castrated NOD/SCID male mice.

We and others have reported that ADT promotes NE phenotypes in prostate cancer cells [3, 20, 54–58]. However, the exact mechanism underlying the induction of neuroendocrine differentiation by ADT is still incompletely understood. We speculated that ADT induces NE markers, at least in part, through downregulating REST, which is a well-established master repressor of NE phenotypes. Indeed, treatment of ADT drug MDV3100 in androgen-dependent LNCaP cells downregulates REST (Fig. 3A). Interestingly, treating LNCaP cells with androgen Dihydrotestosterone (DHT) induces REST (Fig. 3A). Similarly, REST is reduced by MDV3100 in CRPC cells C4-2, and this reduction was rescued by adding DHT along with MDV3100 (Fig. 3B). Culturing prostate cancer cells in media with charcoal-stripped serum (CSS), which is hormone deprived, is another common method to introduce ADT in culture. REST expression is also downregulated when C4-2 cells were cultured in media with CSS (Fig. 3C). Correspondingly, C4-2 cells exhibited cell morphology changes that are reminiscent of NE phenotypes, such as extended neurite spikes and size-reduced cell bodies (Fig. 3D). To examine the impact of ADT on REST expression in vivo, we carried out REST RT-PCR on LNCaP xenograft tumors growing in uncastrated vs castrated male NOD/SCID mice [20]. Castration, i.e., surgical removal of the major androgen-producing organ testis, is a common method of ADT in vivo. As shown in Fig. 3E, REST expression is downregulated by castration. Altogether, these results clearly show that ADT downregulates REST expression.

### Activated CREB1 signaling represses REST

**Fig. 4:**
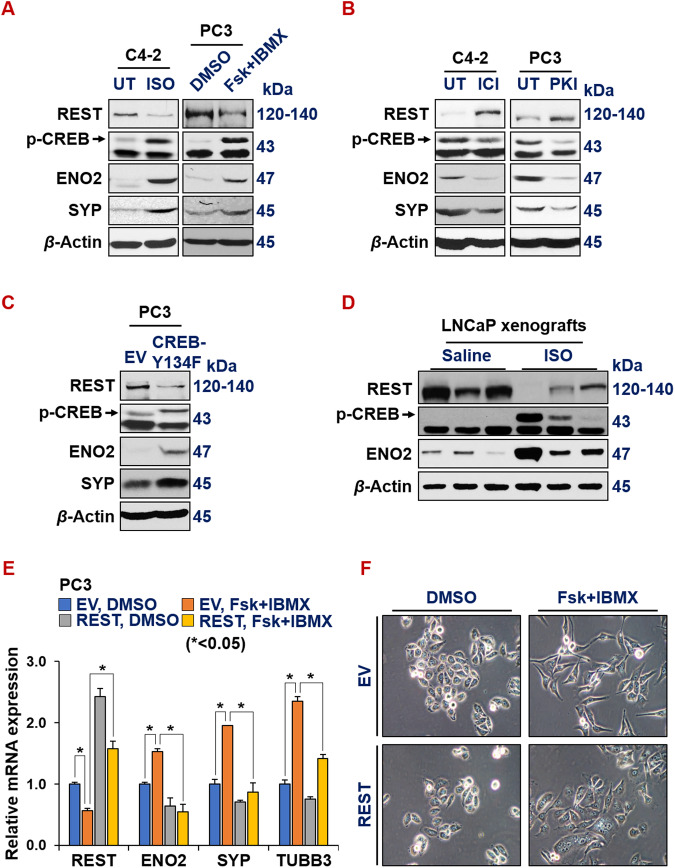
Activated CREB signaling represses REST. Western blotting for REST, p-CREB1 (pS133, indicator of activation) and NE markers in prostate cancer cells upon treatments of CREB1 signaling activator 15 µM isoproterenol (ISO) or 10 µM Forskolin+ 0.5 mM IBMX for 24 h (**A**), or CREB1 signaling inhibitor ICI*-*118,551 (ICI, 10 µM) or synthetic peptide inhibitor of PKA (PKI, 10 µM) for 24 h (**B**). UT: untreated control, because ISO, ICI and PKI were dissolved in water. **C** Western blotting for REST, pS133-CREB1 and NE markers in PC3 cells carrying either an empty vector or CREB1-Y134F cDNA, a constitutively activated form of CREB1. **D** Western blotting for REST, pS133-CREB1, NE markers, EZH2 catalytic product H3K27me3 histone mark, loading controls histone 3 (H3) and beta actin in LNCaP cell-derived xenografts from NOD/SCID male mice, treated with saline or 10 mg/kg ISO for 54 days. **E**, **F** PC3-EV or PC3-REST cells were treated with DMSO control or CREB1 signaling activator combo 10 µM Forskolin (Fsk) + 0.5 mM IBMX for 24 h. mRNA levels of indicted genes were normalized to GAPDH control. **F** Morphology of PC3-EV and PC3-REST cells treated with DMSO or Fsk+IBMX.

We next investigated how ADT downregulates REST transcription in prostate cancer cells. CREB1 signaling and REST are known to antagonize each other in controlling neuronal gene expression in neurobiology [12–14]. Their relationship in prostate cancer cells is unknown. We previously showed that ADT activates CREB1 signaling, which is critical for NED of prostate cancer cells [20]. We speculated that CREB1 signaling downregulates REST expression. To test this hypothesis, we first treated prostate cancer cells with known compounds that either enhance or inhibit CREB1 signaling, followed by examining REST and NE marker expression. Isoproterenol (ISO), an analog of adrenaline, acts as an agonist for beta-adrenergic receptor, enhancing PKA/CREB1 signaling [59, 60]. Additionally, the combination of Forskolin and IBMX (Fsk+IBMX) also activates PKA/CREB1 pathway [61]. Conversely, beta-adrenergic receptor antagonist ICI*-*118,551 (ICI) and synthetic peptide inhibitor of PKA (PKI) are two known inhibitors of PKA/CREB1 signaling. We found that REST levels are reduced, while p-S133-CREB1 (an indicator of CREB1 activation) and NE markers are increased, when prostate cancer cells are treated with ISO or Fsk+IBMX (Fig. 4A, Supplementary Fig. S2A, B). On the contrary, REST is induced, while p-S133-CREB and NE markers are reduced by ICI or PKI (Fig. 4B). When CREB1 signaling is enhanced by overexpressing a constitutively activated form of CREB1 cDNA (i.e., CREB1-Y134F [62]), REST is downregulated while NE markers ENO2 and CHGA are induced in PC3 cells (Fig. 4C and Supplementary Fig. S2C).

To evaluate this CREB1-repression of REST in vivo, we measured protein levels of REST and ENO2 in LNCaP cell-derived xenografts from NOD/SCID male mice treated with saline or 10 mg/kg ISO twice a day for 21 days [63]. As shown in Fig. 4D, REST is downregulated and NE marker ENO2 is induced in LNCaP tumors from the mice treated with ISO. Moreover, we found that REST cDNA overexpression in PC3 cells reversed NE marker induction by the activation of CREB1 signaling by Fsk+IBMX (Fig. 4E), which suggests that REST downregulation is essential for CREB1’s induction of NE markers in prostate cancer cells. Concordantly, the NE-like cell morphology induced by CREB1 activation was abrogated by REST overexpression in PC3 and LNCaP cells (Fig. 4F and Supplementary Fig. S2D respectively). All these results together indicate that CREB1 signaling induces NE markers through repressing REST expression in prostate cancer cells.

### REST is a novel epigenetic target of EZH2 and it reverses EZH2-induction of NE markers

**Fig. 5:**
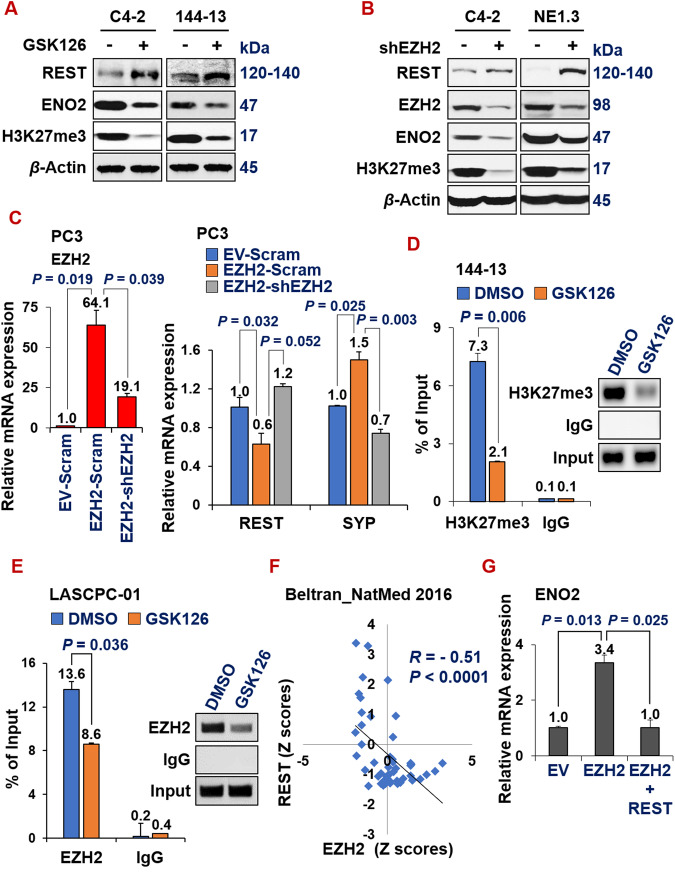
REST is a novel epigenetic target of EZH2, and it reverses EZH2-induction of NE markers. **A** C4-2 and 144-13 cells were treated with DMSO or 10 µM GSK126 for 48 h. Whole cell lysate was analyzed by Western blotting. **B** Western blotting of indicated proteins in C4-2 and NE1.3 cells expressing scramble shRNA or shEZH2. **C** RT-qPCR showing that EZH2-mediated REST downregulation and SYP upregulation was reversed by shEZH2 in PC3 cells (genetic rescue). NEPC cells were treated with DMSO or 10 µM GSK126 for 48 h, followed by ChIP with control IgG, or anti-H3K27me3 antibody (**D**, in 144-13 cells) or anti-EZH2 antibody (**E**, in LASCPC-01 cells), and qPCR of REST’s promoter region near its transcriptional starting site. Y-axis represents % of ChIPed DNA relative to input. **F** Scatter plots showing negative correlation of the expression of EZH2 and REST in the Beltran_NM2016 CRPC genomic dataset. The plot, Pearson correlation coefficient R and P value were directly downloaded from the cBioPortal genomics interface. **G** A genetic rescue experiment and subsequent RT-qPCR in LNCaP cells indicate that the induction of NE marker ENO2 by EZH2 was reversed by additional overexpression of REST.

In the literature, there have been several elegant studies documenting the repression of REST function in prostate cancer through the mechanisms of alternative splicing and protein degradation [64–67]. The transcriptional regulation responsible for REST downregulation in NEPC is still obscure, despite the observation that REST mRNA level is pronouncedly reduced in NEPC (Fig. 1). Therefore, we considered that REST can be downregulated by transcriptional repressors, specifically epigenetic modulators. In a recent study surveying 147 epigenetic regulators in multiple patient datasets, Clermont et al. found that several PRC2 complex proteins, such as EZH2 and CBX2, are among the most upregulated epigenetic regulators in NEPC [41]. We and others have recently implicated the role of EZH2 in regulating NE phenotype in prostate cancer cells [20, 39, 40, 68]. However, the mechanism underlying EZH2’s induction of NE markers is still unclear. We hypothesized that REST downregulation in NEPC is at least partially influenced by the increased activity of the PRC2 complex, and REST is a target of EZH2. Indeed, treating several prostate cancer cell lines with GSK126, an inhibitor of EZH2, resulted in REST induction and reduced NE marker ENO2 expression (Fig. 5A and Supplementary Fig. S3A). Similarly, REST was induced and ENO2 was downregulated upon silencing EZH2 with shRNA (Fig. 5B). In line with these results, overexpressing EZH2 cDNA in PC3 cells downregulated REST expression (Fig. 5C), and this downregulation was rescued by additional expression of shEZH2. NE maker SYP was induced by EZH2 cDNA, which was reversed by additional expression of shEZH2, as expected (Fig. 5C).

To determine whether REST is a direct target of EZH2, we first examined ENCODE ChIP-seq datasets. We found that EZH2 binds to the REST promoter in multiple cell lines and under several conditions (Supplementary Fig. S3B), which suggests that REST is an epigenetic target of EZH2. To confirm this in NEPC cells, we performed chromatin immunoprecipitation (ChIP) using antibodies against H3K27me3, a histone repressive mark primarily catalyzed by EZH2. We carried out H3K27me3 ChIP and qPCR of the DNA sequence on REST promotor in NEPC 144-13 cells treated with DMSO control or EZH2 inhibitor GSK126. As shown in Fig. 5D, there is a clear H3K27me3 mark on the REST promoter in DMSO-treated cells, which is reduced by GSK126. To confirm a direct role of EZH2 protein in binding to REST promoter, we also carried out ChIP using EZH2 antibody. EZH2 binding to REST promoter is reduced by GSK126 in NEPC cells (Fig. 5E). Consistently with REST being a novel EZH2-repressed target in NEPC, they have a significantly negative correlation of expression in two well-cited advanced prostate cancer datasets (Beltran_NatMed [39] in Fig. 5F and SU2C-PCF [52] in Supplementary Fig. S3C). Finally, we set out to determine whether REST downregulation is essential for EZH2’s induction of NE markers, by performing an epistasis experiment (genetic rescue). Of note, overexpression of REST reversed the induction of NE marker ENO2 by EZH2 overexpression in LNCaP cells (Fig. 5G). These results establish that REST is a novel epigenetic target of EZH2, whose repression is critical for EZH2’s induction of NE markers.

### ADT induces NE by downregulating REST through CREB1-activated EZH2 epigenetic repression

**Fig. 6:**
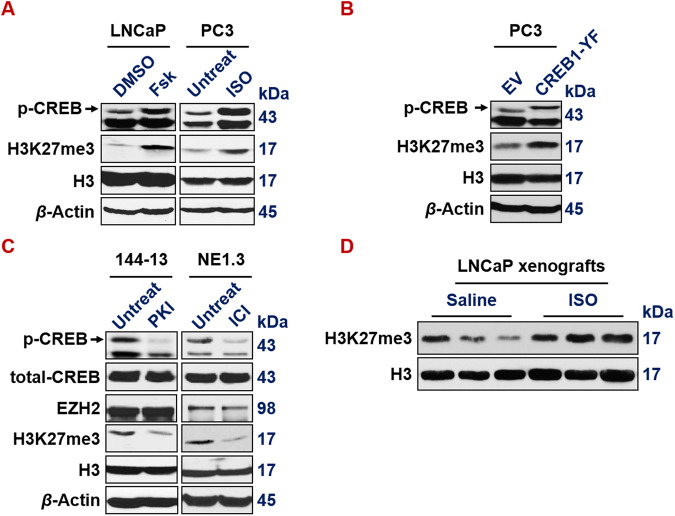
CREB1 signaling enhances EZH2’s PRC2 activity. **A** CREB1 signaling activator Fsk (left) (10 µm for 24 h in LNCaP cells) and with ISO (right) (15 µm for 24 h in PC3 cells) activates p-S133-CREB1 and induces bulk H3K27me3 levels. Histone 3 (H3) and beta actin serve as loading controls for nuclear and whole cell lysate proteins, respectively. **B** p-S133-CREB1 and H3K27me3 levels are induced in PC3 cells expressing constitutively activated CREB1-Y134F mutant cDNA. **C** CREB1 signaling inhibitor PKI (left) (10 µm for 24 h in 144-13 cells) and with ICI (right) (10 µm for 24 h in NE1.3 cells) reduces p-S133-CREB1 and bulk H3K27me3 levels. **D** Western blotting indicates that bulk levels of H3K27me3 mark are elevated in LNCaP xenograft tumors from NOD/SCID male mice that were treated with ISO.

We previously reported that CREB1 signaling leads to the activation of EZH2 which subsequently induces NE markers [20]. Here we confirmed this conclusion, by showing that expressing activated CREB1 cDNA (Y134F), or treatment of CREB1 signaling activator Forskolin or ISO, increases H3K27me3 epigenetic mark in LNCaP and PC3 prostate cancer cells (Fig. 6A, B). Conversely, CREB1 signaling inhibitor PKI or ICI reduces H3K27me3 level in 144-13 and NE1.3 NEPC cells (Fig. 6C). ISO treatment also induces a bulk level of H3K27me3 histone mark in LNCaP mouse xenograft tumors (Fig. 6D).

**Fig. 7:**
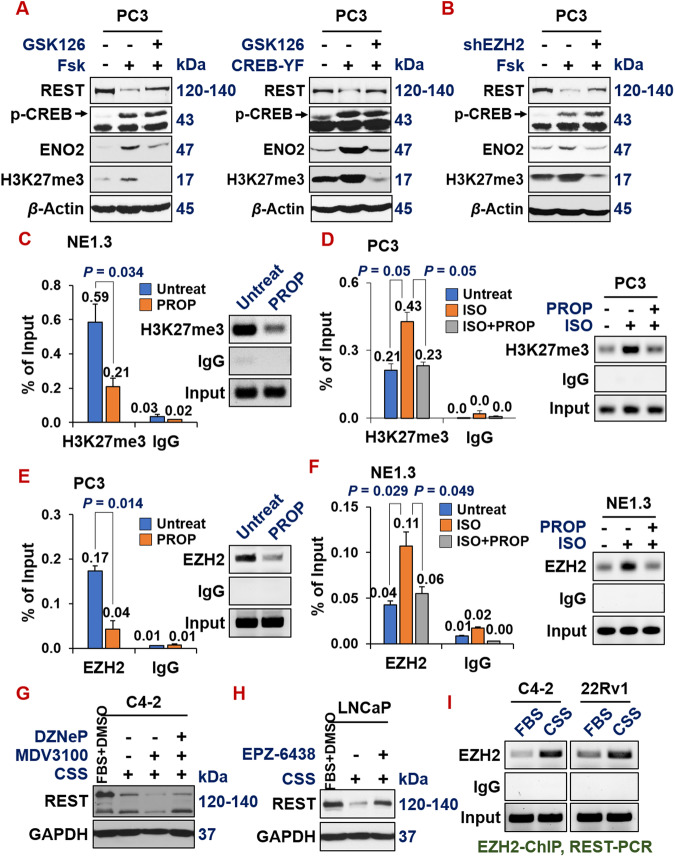
ADT induces NE by downregulating REST through CREB1-activated EZH2 epigenetic repression. **A** Western blotting on PC3 cells shows that REST is downregulated by CREB1 signaling activator forskolin (10 µM, left) or by overexpressing activated CREB1 cDNA (right), which is reversed by EZH2 inhibitor GSK126 (10 µM). **B** Western blotting shows that, when EZH2 is silenced by shEZH2, forskolin could no longer repress REST and induce NE marker ENO2 and H3K27me3 in PC3 cells. ChIP-qPCR measuring H3K27me3 histone mark levels on REST promoter, after treating with indicated CREB1 signaling modulators: propranolol (PROP, 15 µM) for 24 h in NE1.3 (**C**) and PC3 (**D**). ChIP-qPCR measuring EZH2 protein binding on REST promoter upon treating with these CREB1 signaling modulators in PC3 (**E**) and NE1.3 (**F**). **G** C4-2 cells were grown in FBS or CSS media for 7 days, then treated with DMSO or 10 µM MDV3100 for 3 days, followed by treatments of DMSO or 5 µM EZH2 inhibitor DZNeP for 2 days, and then Western blotting of REST and loading control GAPDH. **H** LNCaP cells were grown in FBS or CSS media for 5 days, then treated with DMSO or 10 µM EZH2 inhibitor EZP-6438 for 2 days. Whole cell lysates were collected and analyzed by western blotting for REST and loading control GAPDH. **I** C4-2 and 22Rv1 cells were grown in FBS or CSS media for 7 days and then EZH2 protein binding on the REST promoter region were measured by EZH2 ChIP-qPCR. The quantifications and graphs for these ChIP-PCRs are in Supplementary Fig. S3D.

Given that we have illustrated that REST is a direct target of EZH2 in NEPC cells, next, we set out to determine the genetic relations of the three proteins on this CREB1-EZH2-REST pathway in the context of ADT-induction of NE markers in prostate cancer cells. First, repression of REST by CREB1 activators Forskolin+IBMX is reversed by EZH2 inhibitor GSK126 (Fig. 7A, left) or shEZH2 (Fig. 7B), suggesting that EZH2 is key to the CREB1-mediated repression of REST.

A similar result was observed when CREB1 signaling was activated by expressing constitutively active CREB1 cDNA (Y134F) (Fig. 7A, right). Second, as shown by ChIP-PCR in Fig. 7C, the H3K27me3 histone mark on REST promoter is evidently lower in NE1.3 cells treated with propranolol (PROP), another beta-adrenergic antagonist and an inhibitor of CREB1 signaling [20, 58, 63]. Concordantly, the H3K27me3 histone mark on the REST promoter is induced by CREB1 signaling activator ISO, which is reversed by additional treatment with PROP (Fig. 7D). Similar observations could be made when directly examining EZH2’s binding to REST promoter in these conditions, by performing the ChIP with EZH2 antibody (Fig. 7E, F). Third, when EZH2 activity is inhibited by its inhibitors DZNeP and EPZ-6438 in C4-2 and LNCaP cells respectively, ADT (induced by growing cells in CSS media and treated with MDV300) no longer represses REST expression (Fig. 7G, H). Of note, EZH2 protein’s binding to REST promoter is induced by ADT in AR + C4-2 and 22Rv1 cells (Fig. 7I and Supplementary Fig. S3D). Taken together, these results indicate that ADT induces NE by downregulating REST through CREB1-activated EZH2 epigenetic repression.

In summary, in this study, we have delineated a new critical pathway of CREB1-EZH2-REST, which underlies NE induction by ADT. We showed that REST is downregulated in NEPC cells and patient samples. As expected, REST represses NE markers and prostate cancer progression. Furthermore, ADT downregulates REST both in vitro and in vivo. Notably, ADT-activated CREB1 signaling enhances EZH2’s epigenetic repression of REST, which in turn induces NE markers in prostate cancer cells.

## Discussion

It has been shown by numerous studies that ADT induces NE markers in prostate cancer cells. However, the mechanisms underlying NE induction by ADT are incompletely understood. Here we first showed that ADT induces NE markers by downregulating REST, the master NE repressor. The majority of studies on the regulations of REST expression in cancer have been on its protein degradation or alternative splicing [64–67]. We found that REST mRNA level is pronouncedly reduced in NEPC (Fig. 1). The transcriptional regulation responsible for REST downregulation in NEPC is less clear. We further reported that REST is transcriptionally downregulated by EZH2, which is upregulated in NEPC and activated by CREB1 signaling.

REST and CREB1 have been reported to functionally antagonize each other in regulating neurogenesis [12–14]. For example, Laneve et al. showed that CREB1 induces, while REST reduces, neuronal miRNA miR-9-2 in neuroblastoma cells [12]. However, as far as we know, a direct repression between these two master regulators has not been reported. Our study provides evidence that CREB1 signaling represses REST mRNA and protein expression during neuroendocrine differentiation of prostate cancer cells (Fig. 4). It is worth examining whether this CREB1 repression of REST transcription also occurs in neurobiology.

As to how CREB1 downregulates REST expression, we demonstrated that CREB1 signaling enhances EZH2 activity, which in turn epigenetically represses REST. Interestingly, Laneve *et al* further showed a negative feedback regulation between miR-9-2 and REST [12]. It warrants further investigation to determine whether miR-9-2 induction also contributes to REST repression by CREB1 signaling, besides EZH2’s epigenetic regulation that we demonstrated in this study.

Our study also reveals a surprising relationship between REST and EZH2. EZH2 and REST have been viewed as partners in transcriptional repression in several contexts. It has been reported that REST physically interacts with PRC2 complex proteins EZH2 and SUZ12 [48–50]. Furthermore, ENCODE ChIP datasets showed a significant overlap of target genes between REST and EZH2 or SUZ12. In addition to recruitment by REST, the enzymatic PRC2 core component EZH2 can methylate REST, which leads to the stabilization of REST when bound to the RE1 sites of REST target genes [51]. Therefore, it is counterintuitive that EZH2 represses REST expression, as we have shown in Fig. 5. EZH2 and REST clearly have opposite functions and expression patterns in NEPC compared to prostate adenocarcinoma [20, 39, 40, 68] (Figs. 1, 2 and 5). Therefore, our study introduces a conceptual innovation and context-dependence in the relationship between EZH2 and REST. It remains an interesting question for future investigation regarding how the EZH2-REST relationship evolves during prostate cancer progression, from REST being an EZH2 partner in prostate adenocarcinoma cells to REST becoming an EZH2 target in NEPC cells.

To summarize, we have demonstrated that REST is a novel epigenetically repressed target of EZH2 in NEPC and this EZH2-REST axis is essential for ADT-induced NED. Our results provide critical new insights into the mechanisms underlying NED that is induced by ADT and EZH2 activity. In addition, our findings have revealed a direct antagonistic relationship between two master regulators of neuronal genes in neurobiology, CREB1 and REST. These results not only expand our understanding of prostate cancer progression, they will also forge links among multiple disciplines, including cancer progression, drug resistance, cellular differentiation, epigenetic regulation, and neurobiology.

## Methods

### Cell culture

LNCaP cells and 293 T cells were originally purchased from ATCC. The PC3 prostate cancer cells used in this study represent a poorly metastatic PC3 variant that was kindly provided by Dr. Isaiah Fidler [69] and was matched to PC3 cells from ATCC by DNA STR fingerprinting (Biosynthesis Inc). LNCaP and PC3 cells were maintained in RPMI 1640 media (Mediatech), supplemented with 10% FBS (Gibco) and 1% penicillin-streptomycin. 293 T cells were cultured in DMEM media (Mediatech), supplemented with 10% FBS and 1% penicillin-streptomycin. NEPC NE1.3 cells were derived in Dr. Ming-Fong Lin’s lab from LNCaP cells after long term culturing in charcoal striped serum (CSS) medium [70] and they are cultured in phenol red-free RPMI 1640 medium supplemented with 5% CSS (Gibco) and 1% penicillin and streptomycin. We generated LNCaP-AI (androgen independent) cells in house by culturing LNCaP cells in RPMI 1640 media with CSS for >12 months and are maintained in the same CSS media. LN3 is a LNCaP derivative isolated from in vivo selection of LNCaP cells metastasizing to lymph nodes in NOD/SCID mice [69]. LNCaP, C4-2, LNCaP-AI, LN3 and NE1.3 lines were all matched to ATCC LNCaP profile by DNA STR fingerprinting (Biosynthesis Inc). NEPC cell line LASCPC-01 was obtained from ATCC. NEPC cell line 144-13 were kindly provided by Drs. Sankar Maity and Nora Navone and cultured as described [71]. Cultures were grown in a 37 °C incubator with 5% CO_2_. All cell lines were routinely confirmed to be mycoplasma-free using the Lonza MycoAlert Detection kit (LT07-218).

### In vitro treatments with activators and inhibitors

The activators and inhibitors used in this study were obtained from the following sources: Isoproterenol (ISO) (Sigma), Forskolin (FSK, LC Laborartoy), IBMX (Adipogen), ICI118,551 (ICI) (Tocris), propranolol (PROP) (Tci America), protein kinase A inhibitor peptide 14-22 (PKI) (Tocris), GSK126 (Selleck), EPZ-6438 (Selleck), DZNeP (Apexbio), MDV3100 (Apexbio) and Doxycycline (Enzo). The doses and duration of their treatments were as indicated. ISO, PROP, ICI, PKI and Doxycycline were dissolved in water, while all other chemicals were dissolved in DMSO.

### cDNA/shRNA transduction and transfection

All shRNA constructs are in pLKO.1 vector [72] and were purchased from Sigma-Aldrich (St. Louis, MO). For stable knockdown, cells were transduced with lentiviral particles of Scramble control shRNA: CCTAAGGTTAAGTCGCCCTCG; EZH2 shRNA: CGGAAATCTTAAACCAAGAAT [42, 73]; shREST: GCCTCTAATCAACATGAAGTA. These shRNAs were packaged into viral particles using 293 T cells according to previously described method [72]. Briefly, 293 T cells were seeded in 6-well plates at 1.5-million cells/well. Lentiviral vector carrying shRNA was transfected, together with packaging plasmids VSVG and Delta 8.9, into 293 T cells by TransIT-LT1, followed by centrifugation at 1100 × *g* for 30 min. After initial medium change around 16 h post-transfection, the virus supernatant was collected 48 and 72 h after transfection, aliquoted and stored at −80 °C for subsequent experiments. Cells were infected with the lentivirus supernatant in the presence of 8 μg/ml polybrene and subsequently selected with 1 μg/ml of puromycin. For overexpression of EZH2, cells were infected with retrovirus for human EZH2 cDNA or pBABE-puro vector control [74], and subsequently selected with 1 μg/ml of puromycin. For expressing constitutively active CREB1 mutant, PC3 cells were transfected with pcDNA3-Flag-empty vector (EV), or pcDNA3-CREB-Y134F (YF, constitutively active [62]), kindly provided by Dr. Rebecca Berdeaux, using TransIT-LT1 transfection reagent (Mirus) and selection with 400 μg/ml of G418 for 2 weeks. REST overexpression was achieved by LT1-mediated transfection of pcDNA3-EV, or pcDNA3-REST vector (a generous gift from Dr. Hsing-Jien Kung [18]) and selection with 400 μg/ml of G418 for 2 weeks.

### Reverse transcription and quantitative PCR (RT-qPCR)

Total RNA was extracted by using TRIzol Reagent (Life Technology). The RNA concentration and purity were measured by NanoDrop 2000 UV-Vis Spectrophotometer (Thermo Scientific). 2–3 μg of total RNA was used to generate cDNA using the iScript R Transcription Supermix (Bio-Rad). Real time qPCR was performed using SsoFast EvaGreen Supermix in CFX96 Thermal Cycler (Bio-Rad) or PowerUp SYBR Green Master Mix (Life Technology). PCR-based amplification was performed using the following primers:

REST F: 5′-tggaaaatgcaactatttttcaga-3′; REST R: 5′-gaacttgagtaaggacaaagttcaca-3′. EZH2 F: 5′-ccgctgaggatgtggatac-3′; EZH2 R: 5′-cagtgtgcagcccacaac-3′; CREB F: 5′-ggagcttgtaccaccggtaa-3′; CREB R: 5′-gcatctccactctgctggtt-3′; CHGA F: 5′-tacaaggagatccggaaagg-3′; CHGA R: 5′-ccatctcctcctcctcctct-3′; CHGB F: 5′-cacgccattctgagaagagc-3′; CHGB R: 5′-tctcctggctcttcaaggtg-3′; ENO2 F: 5′-ctgtggtggagcaagagaaa-3′; ENO2 R: 5′-acacccaggatggcattg-3′; SYP F: 5′-ccaatcagatgtagtctggtcagt-3′; SYP R: 5′-aggccttctcctgagctctt-3′. TUBB3 F: 5′-atcagcaaggtgcgtgaggagtat-3′; TUBB3 R: 5′-tcgttgtcgatgcagtaggtc-3′. GAPDH F: 5′-agccacatcgctcagacac-3′; GAPDH R: 5′-gcccaatacgaccaaatcc-3′; Beta Actin F: 5′-ccaaccgcgagaagatga-3′; Beta Actin R: 5′-ccagaggcgtacagggatag-3′; RPS18 F: 5′-ctttgccatcactgccattaag-3′; RPS18 R: 5′-atcacacgttccacctcatc-3′. GAPDH or beta actin were used to normalize RNA input in RT-qPCRs with similar results. The expression levels were calculated according to the comparative CT method (ΔΔCT).

### Western blotting analysis

Cells were washed in ice-cold PBS and lysed in lysis buffer (30 mM Tris, 200 mM NaCl, 1.5 mM MgCl_2_, 0.4 mM EDTA, 20% Glycerol, 1% NP-40, 1 mM DTT) with Complete mini protease inhibitor cocktail and PhosSTOP phosphatase inhibitor cocktail (Roche Applied Science). Protein concentrations were determined using Pierce BCA protein assay kit (Thermo Scientific). The samples were then separated by SDS-PAGE and transferred to PVDF membrane (Bio-Rad). The membrane was blocked with 5% skimmed milk in TBST for 1 h at room temperature, followed by incubation of a primary antibody overnight at 4 °C. The dilutions and catalog numbers of primary antibodies used are listed in Supplementary Table 1. After washes, the membrane was incubated with HRP-conjugated secondary antibodies for 1 h at room temperature. The blots were then detected by Pierce ECL Western Blotting Substrate (Thermo Scientific) on Blue Basic Autoradiography Films (Thomas Scientific).

### Chromatin Immunoprecipitation (ChIP)

DNA binding proteins in cells were cross-linked to DNA by 1% formaldehyde for 10 min at room temperature, which was quenched with glycine. Cells were then lysed in SDS Lysis Buffer (1% SDS, 10 mM EDTA, 50 mM Tris-HCl, pH 8.1 and freshly added protease/phosphatase inhibitors) and sonicated to shear DNA to 300–500 bp fragments using Branson Low Power Ultrasonic Systems 2000 LPt/LPe sonicator (Fisher Scientific). 50 µl of supernatant was diluted in 450 µl dilution buffer (1% Triton X-100, 2 mM EDTA, 20 mM Tris-HCl pH 8.1, 150 mM NaCl supplemented with 0.1% NP40, protease and phosphatase inhibitors). Samples were pre-cleared with protein A/G agrose beads for 2 h. 20 μl of the post-cleared supernatant was kept as input. The remaining supernatants were incubated overnight with 2 µg anti-H3K27me3 (Cell Signaling), EZH2 (Active Motif) or anti-IgG antibody, followed by 1 h incubation with protein A/G agarose beads at 4 °C. The immunoprecipitates were subjected to multiple washes for 5 min each at 4 °C in low salt buffer with 150 mM NaCl, high salt buffer with 500 mM NaCl, LiCl buffer with 250 mM LiCl and finally the TE buffer. DNA was recovered after reversion of the protein-DNA cross-links with 0.2 M NaCl and proteinase K. Subsequently, DNA was extracted with phenol-chloroform and precipitated with ethanol. 5 µl of DNA was subjected to real time PCR. Primers used to measure the enrichment of REST promoter DNA sequence containing H3K27me3 marks are: F (5′-GTGGAAGGGTCTGAAATGGC-3′), and R (5′-GAACTCCCGACTTCTGGTGA-3′). The enrichment of ChIP DNA was calculated as percentage of input. The PCR products were resolved electrophoretically on a 2% agarose gel and visualized by ethidium bromide staining.

### Colony formation

Prostate cancer cells were seeded in a 6-well plate (1000 cells/well) in RPMI 1640 media with 10% FBS and 0.5 mg/ml G418 for continuous selection. The cells were cultured for 14 days. Fresh media with G418 were replenished every 4 days. At day 14, cells were fixed with ice-cold methanol for 10 min and then stained with crystal violet solution for at least 30 min, followed by careful rinsing with sufficient ddH_2_O. After dry, image of the whole 6-well plate was taken.

### MDV3100 treatment and cell viability assay

Cancer cells were seeded in regular RPMI 1640 + 10% FBS media in a 96-well plate (quadruplicate or triplicate). Cells were treated with MDV3100 as indicated for 4 days. AlamarBlue reagent (Thermo Fisher Scientific) was then used to estimate cell numbers in the viability assays, as described [20, 58, 63, 75–77].

### Mouse xenograft tumor experiments

All mouse studies followed protocols approved by the Animal Welfare Committee at the University of Texas Health Science Center at Houston. We have complied with all relevant ethical regulations. Male 6–7-week old NOD/SCID mice were implanted subcutaneously (s.c.) with two million of PC3-EV or PC3-REST cells in 100 µl 1:1 of PBS and Matrigel in both sides of each mouse (5 mice, *n* = 10 tumors for either group). Power calculations indicated that, for analyzing tumor endpoints, at least *n* = 10 per group were needed to assess statistical power of 80% and *P* < 0.05. Mice were randomized in receiving different cell lines or treatments. The investigators carrying out animal experiments were blinded to the group identities when injecting tumor cells or administering treatments. No blinding was practiced upon euthanasia. The mice were sacrificed 8 weeks after the cell implantation. At sacrifice, the s.c. tumors were extracted, weighted and photographed. The tumors were then either formalin-fixed paraffin-embedded or snap-frozen. No samples or animals were regarded as outliers and excluded.

### Genomics data mining

All non-TCGA and TCGA datasets indicated genes were downloaded from cBioPortal [78] and the GEO database (http://www.ncbi.nlm.nih.gov/gds). The transformed and normalized gene expression values from these sources were used in our analysis and statistical calculation.

### Statistical analyses

All experiments were carried out three times, unless indicated otherwise. Statistical analyses were performed using GraphPad software and/or online statistics tools. *P* values were obtained through Student *t* test with two tails and assumed unequal variance, unless otherwise indicated. Spearman correlation coefficient and associated *P* values for gene expression were calculated using GraphPad or a statistics tool at http://vassarstats.net/corr_rank.html and confirmed by another online tool: http://www.socscistatistics.com/tests/spearman/default2.aspx. Student t-tests were carried out in Excel (2-sided, assuming unequal variances). *P* < 0.05 are considered significant. All error bars are defined as s.d. All central values are defined as mean.

### Supplementary information

Suppl. legend

Suppl. Figure S1

Suppl. Figure S2

Suppl. Figure S3

Suppl. Table 1

Western blot -uncropped figures

## Data Availability

The authors declare that the data supporting the findings of this study are available within the article and its supplementary information files, or are available upon reasonable requests to the authors.

## References

[1] Davies A, Zoubeidi A, Selth LA (2020). The epigenetic and transcriptional landscape of neuroendocrine prostate cancer. Endocr Relat Cancer.

[2] Quintanal-Villalonga A, Chan JM, Yu HA, Pe’er D, Sawyers CL, Sen T (2020). Lineage plasticity in cancer: a shared pathway of therapeutic resistance. Nat Rev Clin Oncol..

[3] Davies AH, Beltran H, Zoubeidi A (2018). Cellular plasticity and the neuroendocrine phenotype in prostate cancer. Nat Rev Urol..

[4] Aparicio A, Logothetis CJ, Maity SN (2011). Understanding the lethal variant of prostate cancer: power of examining extremes. Cancer Discov.

[5] Beltran H, Tomlins S, Aparicio A, Arora V, Rickman D, Ayala G (2014). Aggressive variants of castration-resistant prostate cancer. Clin Cancer Res.

[6] Aggarwal R, Huang J, Alumkal JJ, Zhang L, Feng FY, Thomas GV (2018). Clinical and genomic characterization of treatment-emergent small-cell neuroendocrine prostate cancer: a multi-institutional prospective study. J Clin Oncol.

[7] Puca L, Vlachostergios PJ, Beltran H (2019). Neuroendocrine differentiation in prostate cancer: emerging biology, models, and therapies. Cold Spring Harb Perspect Med.

[8] Kaarijarvi R, Kaljunen H, Ketola K (2021). Molecular and functional links between neurodevelopmental processes and treatment-induced neuroendocrine plasticity in prostate cancer progression. Cancers.

[9] Beltran H, Tagawa ST, Park K, MacDonald T, Milowsky MI, Mosquera JM (2012). Challenges in recognizing treatment-related neuroendocrine prostate cancer. J Clin Oncol.

[10] Hirano D, Okada Y, Minei S, Takimoto Y, Nemoto N (2004). Neuroendocrine differentiation in hormone refractory prostate cancer following androgen deprivation therapy. Eur Urol.

[11] Papandreou CN, Daliani DD, Thall PF, Tu SM, Wang X, Reyes A (2002). Results of a phase II study with doxorubicin, etoposide, and cisplatin in patients with fully characterized small-cell carcinoma of the prostate. J Clin Oncol.

[12] Laneve P, Gioia U, Andriotto A, Moretti F, Bozzoni I, Caffarelli E (2010). A minicircuitry involving REST and CREB controls miR-9-2 expression during human neuronal differentiation. Nucleic Acids Res.

[13] Qureshi IA, Gokhan S, Mehler MF (2010). REST and CoREST are transcriptional and epigenetic regulators of seminal neural fate decisions. Cell Cycle.

[14] Wu J, Xie X (2006). Comparative sequence analysis reveals an intricate network among REST, CREB and miRNA in mediating neuronal gene expression. Genome Biol.

[15] Negrini S, Prada I, D’Alessandro R, Meldolesi J (2013). REST: an oncogene or a tumor suppressor?. Trends Cell Biol.

[16] Liang H, Studach L, Hullinger RL, Xie J, Andrisani OM (2014). Down-regulation of RE-1 silencing transcription factor (REST) in advanced prostate cancer by hypoxia-induced miR-106b~25. Exp Cell Res.

[17] Svensson C, Ceder J, Iglesias-Gato D, Chuan YC, Pang ST, Bjartell A (2014). REST mediates androgen receptor actions on gene repression and predicts early recurrence of prostate cancer. Nucleic Acids Res.

[18] Lin TP, Chang YT, Lee SY, Campbell M, Wang TC, Shen SH (2016). REST reduction is essential for hypoxia-induced neuroendocrine differentiation of prostate cancer cells by activating autophagy signaling. Oncotarget.

[19] Chang YT, Lin TP, Campbell M, Pan CC, Lee SH, Lee HC (2017). REST is a crucial regulator for acquiring EMT-like and stemness phenotypes in hormone-refractory prostate cancer. Sci Rep.

[20] Zhang Y, Zheng D, Zhou T, Song H, Hulsurkar M, Su N (2018). Androgen deprivation promotes neuroendocrine differentiation and angiogenesis through CREB-EZH2-TSP1 pathway in prostate cancers. Nat Commun.

[21] Deng X, Liu H, Huang J, Cheng L, Keller ET, Parsons SJ (2008). Ionizing radiation induces prostate cancer neuroendocrine differentiation through interplay of CREB and ATF2: implications for disease progression. Cancer Res.

[22] Suarez CD, Deng X, Hu CD (2014). Targeting CREB inhibits radiation-induced neuroendocrine differentiation and increases radiation-induced cell death in prostate cancer cells. Am J Cancer Res.

[23] Hu CD, Choo R, Huang J (2015). Neuroendocrine differentiation in prostate cancer: a mechanism of radioresistance and treatment failure. Front Oncol.

[24] Cao Q, Yu J, Dhanasekaran SM, Kim JH, Mani RS, Tomlins SA (2008). Repression of E-cadherin by the polycomb group protein EZH2 in cancer. Oncogene.

[25] Cha TL, Zhou BP, Xia W, Wu Y, Yang CC, Chen CT (2005). Akt-mediated phosphorylation of EZH2 suppresses methylation of lysine 27 in histone H3. Science.

[26] Wang L, Jin Q, Lee JE, Su IH, Ge K (2010). Histone H3K27 methyltransferase Ezh2 represses Wnt genes to facilitate adipogenesis. Proc Natl Acad Sci USA.

[27] Yamaguchi H, Hung MC (2014). Regulation and role of EZH2 in Cancer. Cancer Res Treat.

[28] Cao R, Wang L, Wang H, Xia L, Erdjument-Bromage H, Tempst P (2002). Role of histone H3 lysine 27 methylation in Polycomb-group silencing. Science.

[29] Simon JA, Lange CA (2008). Roles of the EZH2 histone methyltransferase in cancer epigenetics. Mutat Res.

[30] Vire E, Brenner C, Deplus R, Blanchon L, Fraga M, Didelot C (2006). The polycomb group protein EZH2 directly controls DNA methylation. Nature.

[31] Crea F, Fornaro L, Bocci G, Sun L, Farrar WL, Falcone A (2012). EZH2 inhibition: targeting the crossroad of tumor invasion and angiogenesis. Cancer Metastasis Rev.

[32] Friedman JM, Liang G, Liu CC, Wolff EM, Tsai YC, Ye W (2009). The putative tumor suppressor microRNA-101 modulates the cancer epigenome by repressing the polycomb group protein EZH2. Cancer Res.

[33] Kikuchi J, Takashina T, Kinoshita I, Kikuchi E, Shimizu Y, Sakakibara-Konishi J (2012). Epigenetic therapy with 3-deazaneplanocin A, an inhibitor of the histone methyltransferase EZH2, inhibits growth of non-small cell lung cancer cells. Lung Cancer.

[34] Pal B, Bouras T, Shi W, Vaillant F, Sheridan JM, Fu N (2013). Global changes in the mammary epigenome are induced by hormonal cues and coordinated by Ezh2. Cell Rep.

[35] Shin YJ, Kim JH (2012). The role of EZH2 in the regulation of the activity of matrix metalloproteinases in prostate cancer cells. PloS one.

[36] Varambally S, Cao Q, Mani RS, Shankar S, Wang X, Ateeq B (2008). Genomic loss of microRNA-101 leads to overexpression of histone methyltransferase EZH2 in cancer. Science.

[37] Wan L, Li X, Shen H, Bai X (2013). Quantitative analysis of EZH2 expression and its correlations with lung cancer patients’ clinical pathological characteristics. Clin Transl Oncol.

[38] Yang YA, Yu J (2013). EZH2, an epigenetic driver of prostate cancer. Protein Cell.

[39] Beltran H, Prandi D, Mosquera JM, Benelli M, Puca L, Cyrta J (2016). Divergent clonal evolution of castration-resistant neuroendocrine prostate cancer. Nat. Med.

[40] Ku SY, Rosario S, Wang Y, Mu P, Seshadri M, Goodrich ZW (2017). Rb1 and Trp53 cooperate to suppress prostate cancer lineage plasticity, metastasis, and antiandrogen resistance. Science.

[41] Clermont PL, Lin D, Crea F, Wu R, Xue H, Wang Y (2015). Polycomb-mediated silencing in neuroendocrine prostate cancer. Clin Epigenetics.

[42] Xu K, Wu ZJ, Groner AC, He HH, Cai C, Lis RT (2012). EZH2 oncogenic activity in castration-resistant prostate cancer cells is Polycomb-independent. Science.

[43] Zhao JC, Yu J, Runkle C, Wu L, Hu M, Wu D (2012). Cooperation between Polycomb and androgen receptor during oncogenic transformation. Genome Res.

[44] Beltran H, Rickman DS, Park K, Chae SS, Sboner A, MacDonald TY (2011). Molecular characterization of neuroendocrine prostate cancer and identification of new drug targets. Cancer Discov.

[45] Rockowitz S, Lien WH, Pedrosa E, Wei G, Lin M, Zhao K (2014). Comparison of REST cistromes across human cell types reveals common and context-specific functions. PLoS Comput Biol.

[46] Erkek S, Johann PD, Finetti MA, Drosos Y, Chou HC, Zapatka M (2019). Comprehensive analysis of chromatin states in atypical Teratoid/Rhabdoid Tumor Identifies Diverging Roles for SWI/SNF and polycomb in gene regulation. Cancer Cell.

[47] Qadeer ZA, Valle-Garcia D, Hasson D, Sun Z, Cook A, Nguyen C (2019). ATRX in-frame fusion neuroblastoma is sensitive to EZH2 inhibition via modulation of neuronal gene signatures. Cancer Cell.

[48] Dietrich N, Lerdrup M, Landt E, Agrawal-Singh S, Bak M, Tommerup N (2012). REST-mediated recruitment of polycomb repressor complexes in mammalian cells. PLoS Genet.

[49] Mozzetta C, Pontis J, Fritsch L, Robin P, Portoso M, Proux C (2014). The histone H3 lysine 9 methyltransferases G9a and GLP regulate polycomb repressive complex 2-mediated gene silencing. Mol Cell.

[50] Tsai MC, Manor O, Wan Y, Mosammaparast N, Wang JK, Lan F (2010). Long noncoding RNA as modular scaffold of histone modification complexes. Science.

[51] Lee SW, Oh YM, Lu YL, Kim WK, Yoo AS (2018). MicroRNAs overcome cell fate barrier by reducing EZH2-Controlled REST Stability during neuronal conversion of human adult fibroblasts. Dev Cell.

[52] Abida W, Cyrta J, Heller G, Prandi D, Armenia J, Coleman I (2019). Genomic correlates of clinical outcome in advanced prostate cancer. Proc Natl Acad Sci USA.

[53] Tai S, Sun Y, Squires JM, Zhang H, Oh WK, Liang CZ (2011). PC3 is a cell line characteristic of prostatic small cell carcinoma. Prostate.

[54] Chen WY, Zeng T, Wen YC, Yeh HL, Jiang KC, Chen WH (2019). Androgen deprivation-induced ZBTB46-PTGS1 signaling promotes neuroendocrine differentiation of prostate cancer. Cancer Lett.

[55] Dang Q, Li L, Xie H, He D, Chen J, Song W (2015). Anti-androgen enzalutamide enhances prostate cancer neuroendocrine (NE) differentiation via altering the infiltrated mast cells -> androgen receptor (AR) -> miRNA32 signals. Mol Oncol..

[56] Niu Y, Guo C, Wen S, Tian J, Luo J, Wang K (2018). ADT with antiandrogens in prostate cancer induces adverse effect of increasing resistance, neuroendocrine differentiation and tumor metastasis. Cancer Lett.

[57] Yuan TC, Veeramani S, Lin MF (2007). Neuroendocrine-like prostate cancer cells: neuroendocrine transdifferentiation of prostate adenocarcinoma cells. Endocr Relat Cancer.

[58] Sang M, Hulsurkar M, Zhang X, Song H, Zheng D, Zhang Y (2016). GRK3 is a direct target of CREB activation and regulates neuroendocrine differentiation of prostate cancer cells. Oncotarget.

[59] de Graaf C, Rognan D (2008). Selective structure-based virtual screening for full and partial agonists of the beta2 adrenergic receptor. J Med Chem.

[60] Dishy V, Sofowora GG, Xie HG, Kim RB, Byrne DW, Stein CM (2001). The effect of common polymorphisms of the beta2-adrenergic receptor on agonist-mediated vascular desensitization. N. Engl J Med.

[61] Heisler S, Reisine T (1984). Forskolin stimulates adenylate cyclase activity, cyclic AMP accumulation, and adrenocorticotropin secretion from mouse anterior pituitary tumor cells. J Neurochem.

[62] Du K, Asahara H, Jhala US, Wagner BL, Montminy M (2000). Characterization of a CREB gain-of-function mutant with constitutive transcriptional activity in vivo. Mol Cell Biol.

[63] Hulsurkar M, Li Z, Zhang Y, Li X, Zheng D, Li W (2017). Beta-adrenergic signaling promotes tumor angiogenesis and prostate cancer progression through HDAC2-mediated suppression of thrombospondin-1. Oncogene.

[64] Li Y, Donmez N, Sahinalp C, Xie N, Wang Y, Xue H, et al. SRRM4 Drives neuroendocrine transdifferentiation of prostate adenocarcinoma under androgen receptor pathway inhibition. Eur Urol. 2017;71:68–78. 10.1016/j.eururo.2016.04.028 27180064

[65] Wagoner MP, Gunsalus KT, Schoenike B, Richardson AL, Friedl A, Roopra A (2010). The transcription factor REST is lost in aggressive breast cancer. PLoS Genet.

[66] Westbrook TF, Hu G, Ang XL, Mulligan P, Pavlova NN, Liang A (2008). SCFbeta-TRCP controls oncogenic transformation and neural differentiation through REST degradation. Nature.

[67] Zhang X, Coleman IM, Brown LG, True LD, Kollath L, Lucas JM (2015). SRRM4 expression and the loss of REST activity may promote the emergence of the neuroendocrine phenotype in castration-resistant prostate cancer. Clin Cancer Res.

[68] Mu P, Zhang Z, Benelli M, Karthaus WR, Hoover E, Chen CC (2017). SOX2 promotes lineage plasticity and antiandrogen resistance in TP53- and RB1-deficient prostate cancer. Science.

[69] Pettaway CA, Pathak S, Greene G, Ramirez E, Wilson MR, Killion JJ (1996). Selection of highly metastatic variants of different human prostatic carcinomas using orthotopic implantation in nude mice. Clinical cancer research : an official journal of the American Association for. Cancer Res.

[70] Yuan TC, Veeramani S, Lin FF, Kondrikou D, Zelivianski S, Igawa T (2006). Androgen deprivation induces human prostate epithelial neuroendocrine differentiation of androgen-sensitive LNCaP cells. Endocr Relat Cancer.

[71] Kleb B, Estecio MR, Zhang J, Tzelepi V, Chung W, Jelinek J (2016). Differentially methylated genes and androgen receptor re-expression in small cell prostate carcinomas. Epigenetics.

[72] Moffat J, Grueneberg DA, Yang X, Kim SY, Kloepfer AM, Hinkle G (2006). A lentiviral RNAi library for human and mouse genes applied to an arrayed viral high-content screen. Cell.

[73] Kim KH, Kim W, Howard TP, Vazquez F, Tsherniak A, Wu JN (2015). SWI/SNF-mutant cancers depend on catalytic and non-catalytic activity of EZH2. Nat Med.

[74] Pearlberg J, Degot S, Endege W, Park J, Davies J, Gelfand E (2005). Screens using RNAi and cDNA expression as surrogates for genetics in mammalian tissue culture cells. Cold Spring Harb Symp Quant Biol.

[75] Li W, Ai N, Wang S, Bhattacharya N, Vrbanac V, Collins M (2014). GRK3 is essential for metastatic cells and promotes prostate tumor progression. Proc Natl Acad Sci USA.

[76] Li L, Su N, Zhou T, Zheng D, Wang Z, Chen H (2018). Mixed lineage kinase ZAK promotes epithelial-mesenchymal transition in cancer progression. Cell Death Dis.

[77] Wang Z, Hulsurkar M, Zhuo L, Xu J, Yang H, Naderinezhad S (2021). CKB inhibits epithelial-mesenchymal transition and prostate cancer progression by sequestering and inhibiting AKT activation. Neoplasia.

[78] Cerami E, Gao J, Dogrusoz U, Gross BE, Sumer SO, Aksoy BA (2012). The cBio cancer genomics portal: an open platform for exploring multidimensional cancer genomics data. Cancer Discov.

