# Supplementary material for: Androgen deprivation induces neuroendocrine phenotypes in prostate cancer cells through CREB1/EZH2-mediated downregulation of REST
Source: Cell Death Discov. 2024 May 22;10:246. doi: 10.1038/s41420-024-02031-1 (PMC11111810; doi:10.1038/s41420-024-02031-1)
Supplement: Supplementary file 1 — Suppl. legend [file 41420_2024_2031_MOESM1_ESM.docx]

**Supplementary Fig. S1. Expression correlations of REST with several NE markers in CRPC patient samples.**

Scatter plots, Pearson correlation coefficients and P values between REST and NE markers ENO2, CHGA, CHGB and TUBB3, in SU2C-PCF CRPC samples^53^ were accessed and downloaded from cBioPortal cancer genomics user interface (cbioportal.org).

**Supplementary Fig. S2. Activated CREB signaling represses REST.**

**(A-B)** RT-qPCR of REST and NE markers in prostate cancer cells treated with CREB1 signaling activator combo 10uM Fsk+ 0.5mM IMBX (A, PC3 cells) or 15uM ISO (B, C4-2 cells), for 24hr. **(C)** Differences in mRNA levels of REST and NE markers in PC3-EV and PC3-CREB1-Y134F cells expressing constitutively activated CREB1. **(D)** Morphology of LNCaP-EV and LNCaP-REST cDNA cells treated with DMSO control or 10uM Fsk+ 0.5mM IBMX, for 24hr. REST suppresses NE cell morphology (e.g. long cellular processes and small cell body) that is induced by Fsk+IBMX.

**Supplementary Fig. S3. REST is an epigenetic target of EZH2.**

**(A)** REST is induced, while NE markers ENO2 and CHGA are reduced, by EZH2 inhibitor GSK126 in NEPC cells 144-13 (10uM for 48hr). **(B)** Mining the ENCODE ChIP-seq database reveals that EZH2 binds to REST transcriptional starting site regions in multiple cell lines and several conditions, suggesting that REST is an epigenetic target of EZH2. **(C)** Scatter plots showing negative correlation of the expression of EZH2 and REST in the SU2C-PCF CRPC genomic dataset with NE information. The plot, Pearson correlation coefficient R and P value were directly downloaded from the cBioPortal genomics interface. **(D)** EZH2 ChIP and REST promoter qPCR in C4-2 and 22Rv1 cells growing in CSS media vs FBS media. Y-axis represents % of ChIPed DNA relative to input.
