# Supplementary figures and images for: Androgen deprivation induces neuroendocrine phenotypes in prostate cancer cells through CREB1/EZH2-mediated downregulation of REST
Source: Cell Death Discov. 2024 May 22;10:246. doi: 10.1038/s41420-024-02031-1 (PMC11111810; doi:10.1038/s41420-024-02031-1)

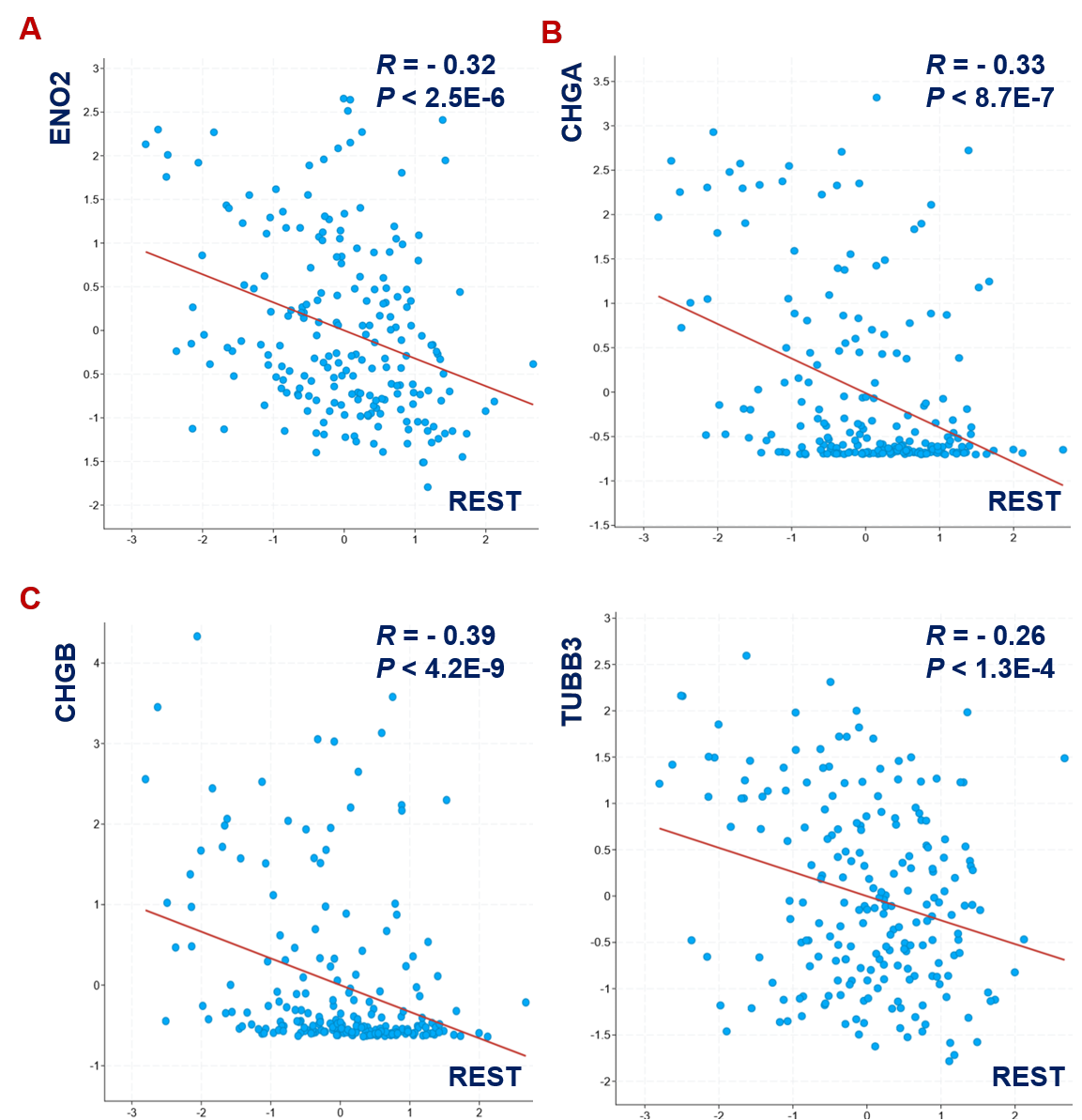

Supplement: Supplementary file 2 — Suppl. Figure S1 [file 41420_2024_2031_MOESM2_ESM.tif]

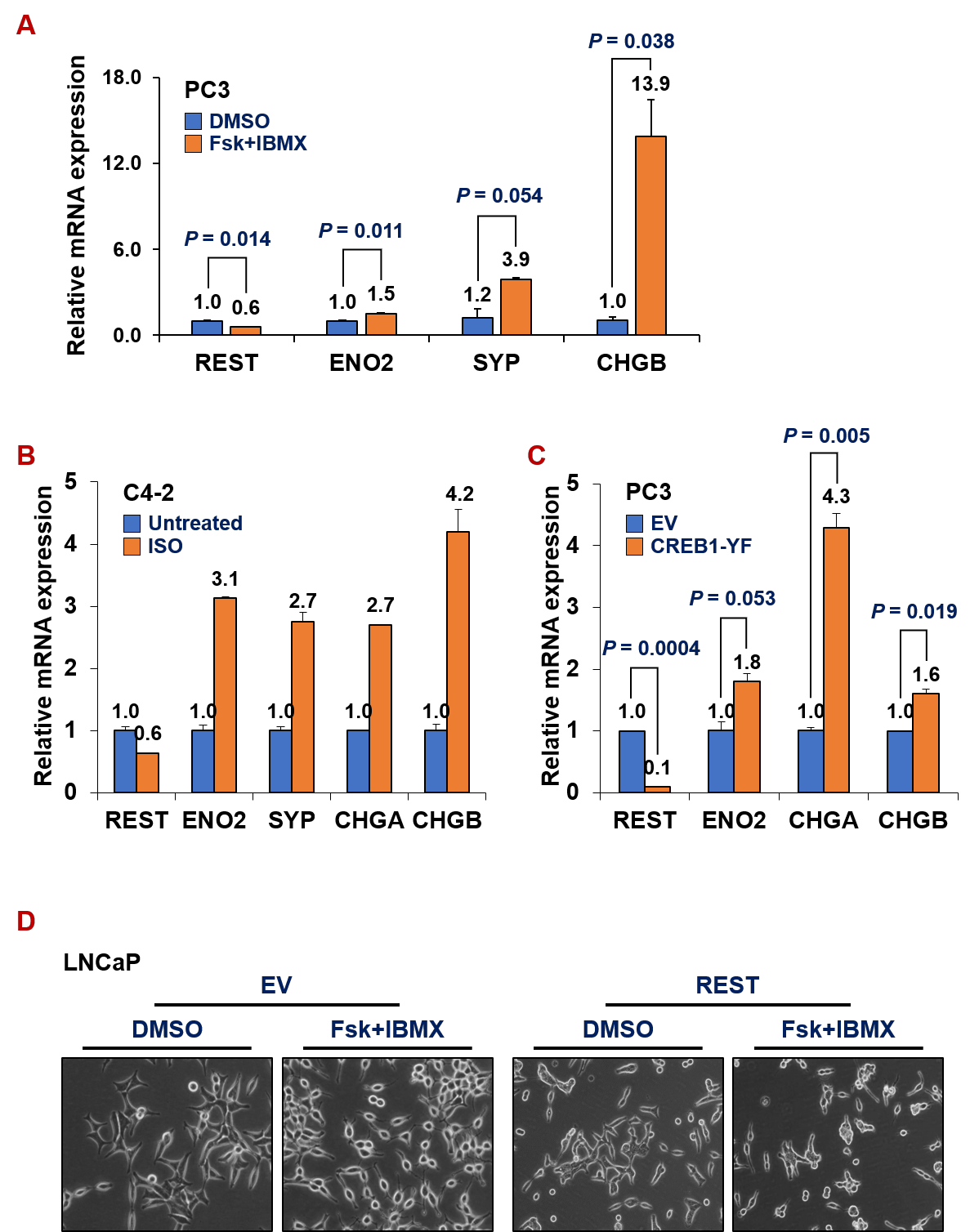

Supplement: Supplementary file 3 — Suppl. Figure S2 [file 41420_2024_2031_MOESM3_ESM.tif]

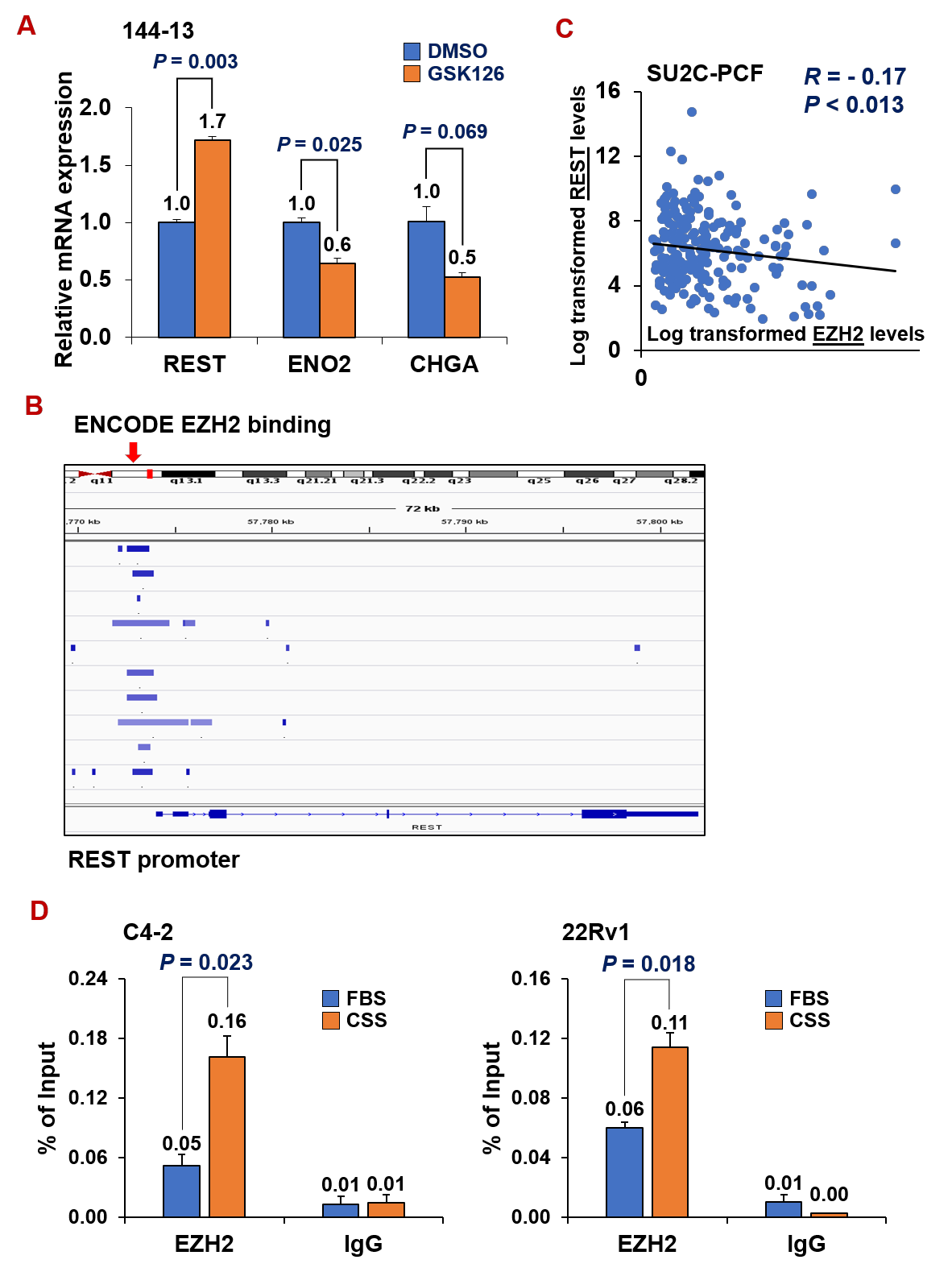

Supplement: Supplementary file 4 — Suppl. Figure S3 [file 41420_2024_2031_MOESM4_ESM.tif]
