# Supplementary material for: Androgen deprivation induces neuroendocrine phenotypes in prostate cancer cells through CREB1/EZH2-mediated downregulation of REST
Source: Cell Death Discov. 2024 May 22;10:246. doi: 10.1038/s41420-024-02031-1 (PMC11111810; doi:10.1038/s41420-024-02031-1)
Supplement: Supplementary file 5 — Suppl. Table 1 [file 41420_2024_2031_MOESM5_ESM.docx]

**Supplementary Table S1.** **Primary antibodies used.**

| **Primary antibody** | **Company** | **Catalog number** |
| --- | --- | --- |
| REST Rabbit Polyclonal | Millipore Sigma | 07-579-MI |
| REST Rabbit Polyclonal | Millipore Sigma | 09-019-MI |
| REST Mouse monoclonal | Millipore Sigma | MABC1560-25 |
| Mouse EZH2 | Active Motif | 39875 |
| Rabbit EZH2 (D2C9) | Cell Signaling | 5246 |
| Rabbit ENO2 | GenScript | A01412 |
| Mouse SYP (D-4) | Santa Cruz | sc-17750 |
| Mouse AR (441) | Cell Signaling | sc-7305 |
| Rabbit H3K27me3 (C36B11) | Cell Signaling | 9733 |
| Mouse H3 (96C10) | Cell Signaling | 3638 |
| Rabbit pS133-CREB1 (87G3) | Cell Signaling | 9198 |
| Mouse CREB1 (86B10) | Cell Signaling | 9104 |
| Mouse Beta Actin | Cell Signaling | 3700 |
| Rabbit GAPDH (14C10) | Cell Signaling | 2118 |
|  |  |  |
|  |  |  |
