# Supplementary material for: Androgen deprivation induces neuroendocrine phenotypes in prostate cancer cells through CREB1/EZH2-mediated downregulation of REST
Source: Cell Death Discov. 2024 May 22;10:246. doi: 10.1038/s41420-024-02031-1 (PMC11111810; doi:10.1038/s41420-024-02031-1)

Appendix Fig. 1

Fig. 1 A

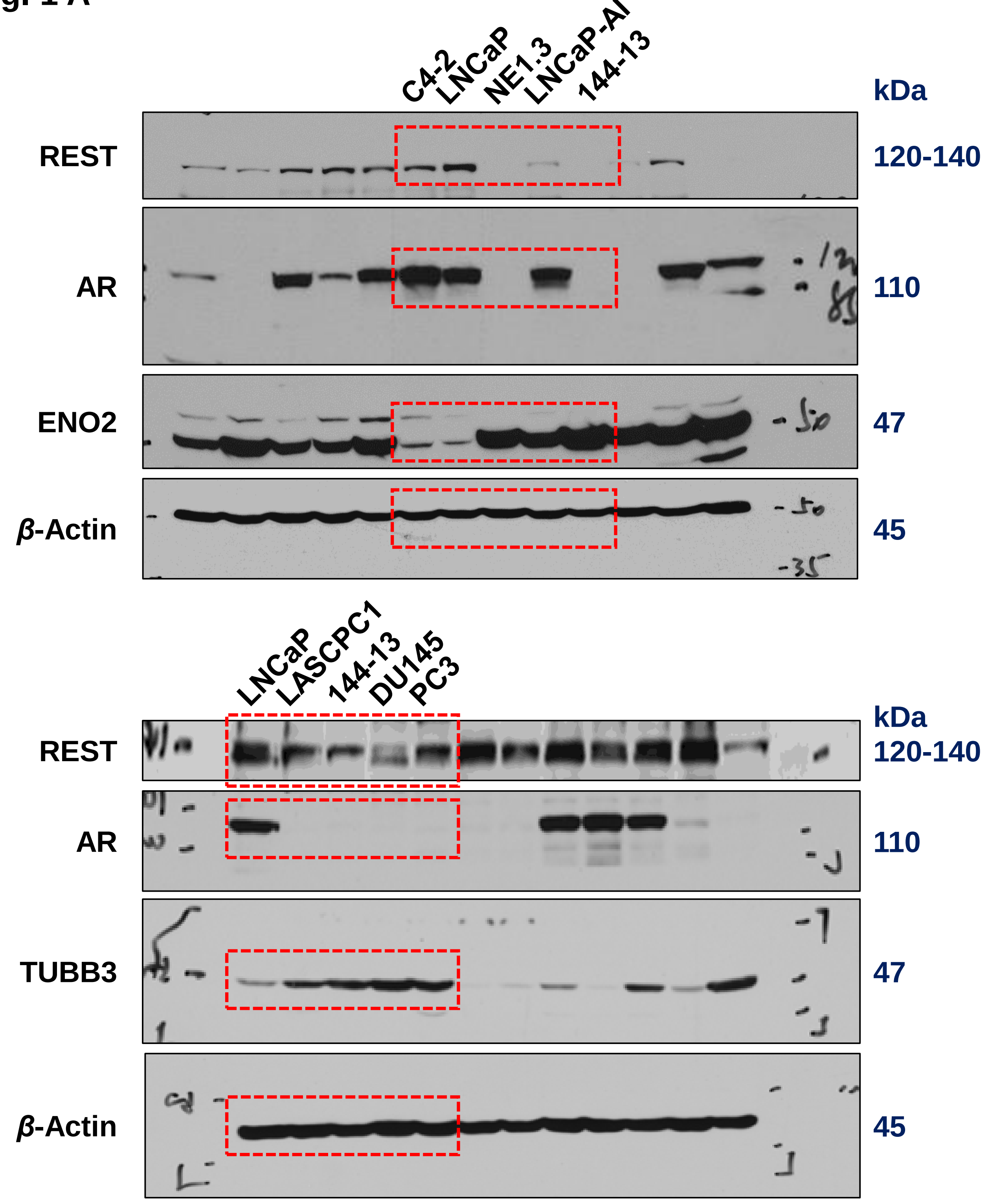

Appendix Fig. 2

Fig. 2 A

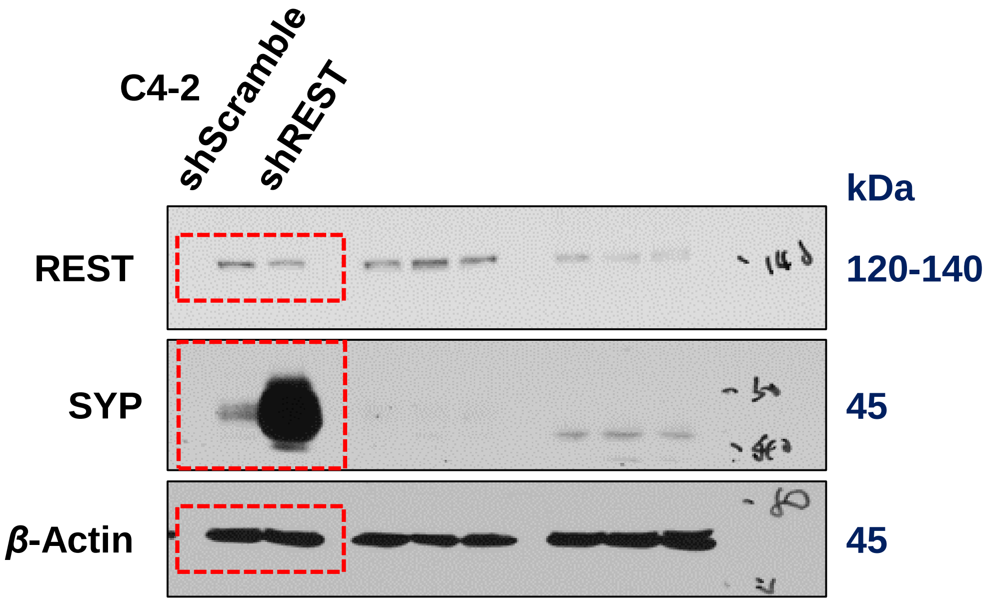

Fig. 2 B

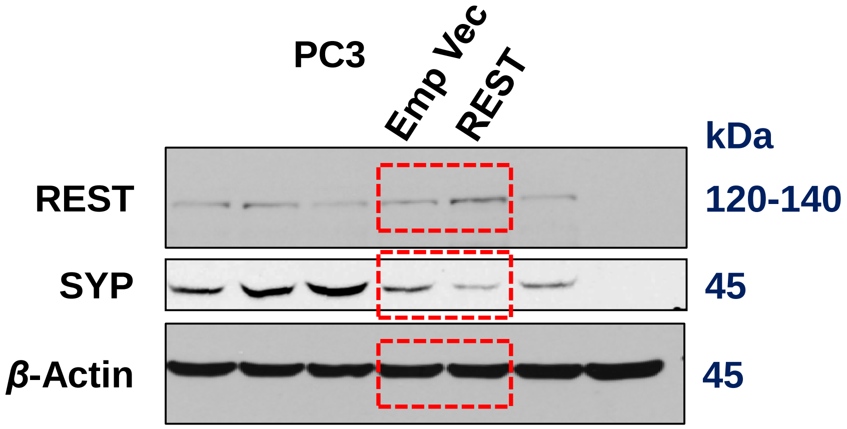

Fig. 2 E

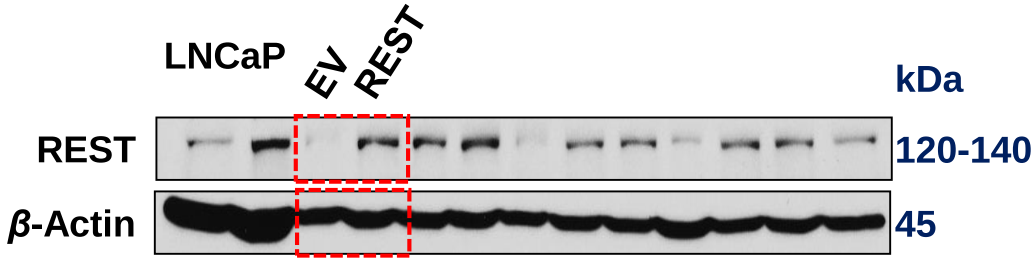

Fig. 2 G

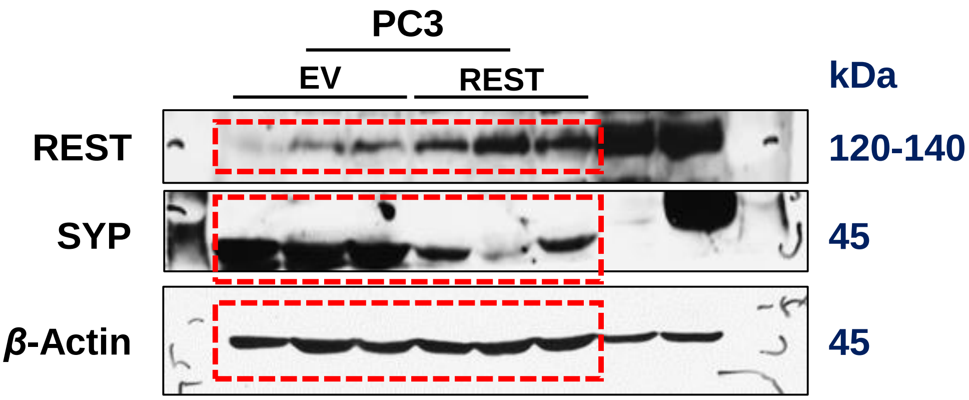

Appendix Fig. 3

Fig. 3 A

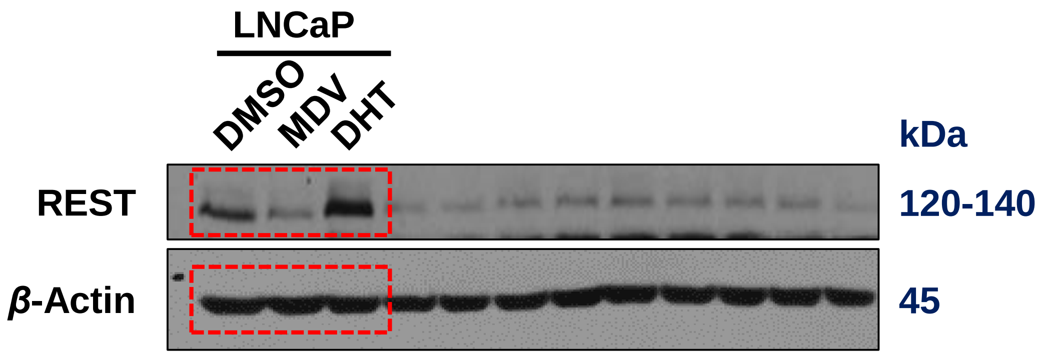

Fig. 3 B

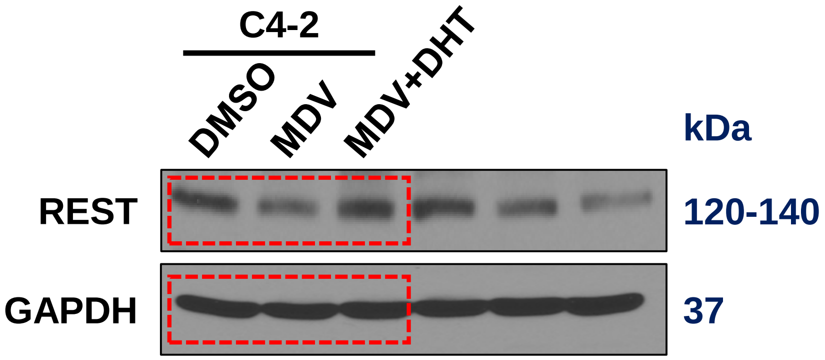

Fig. 3 G

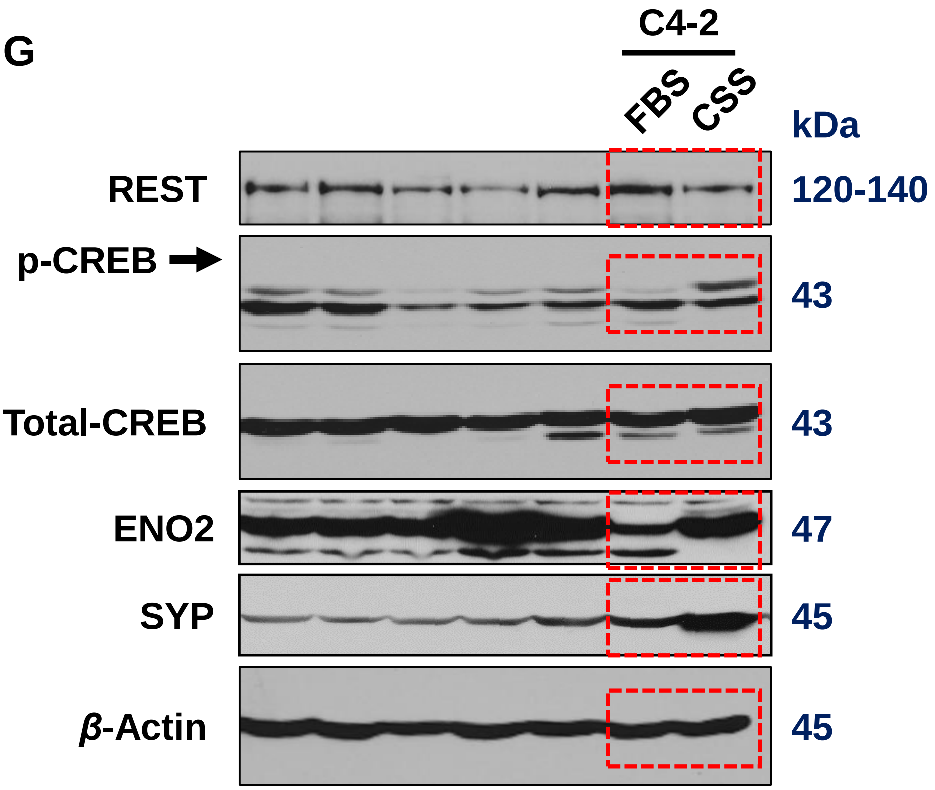

Appendix Fig. 4

Fig. 4 A

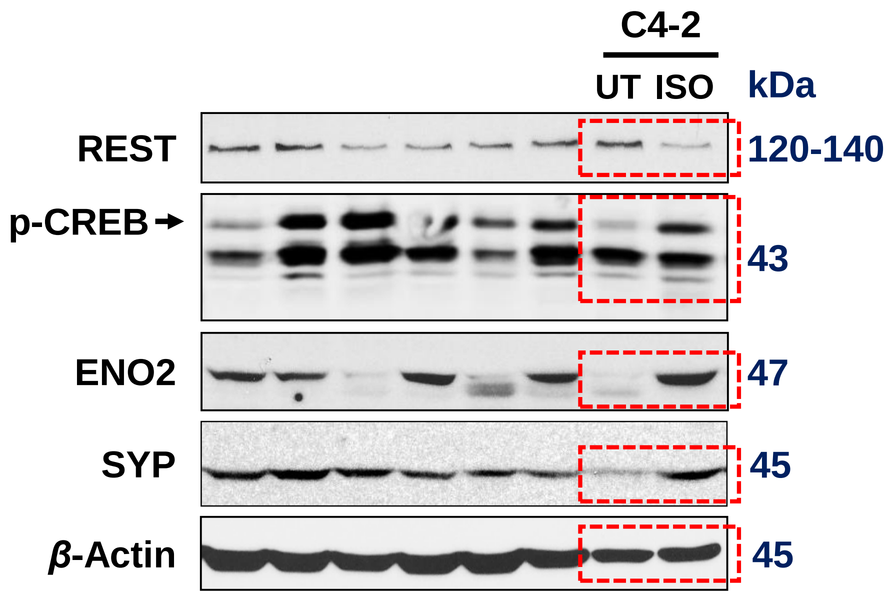

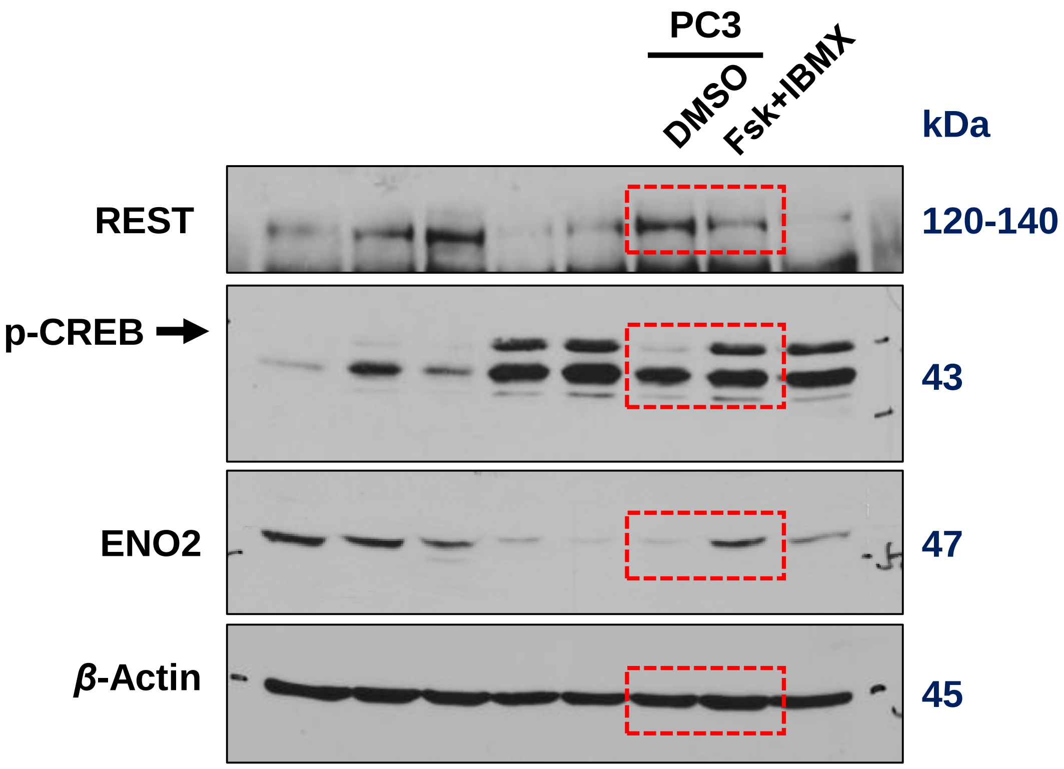

Appendix Fig. 4

Fig. 4 B

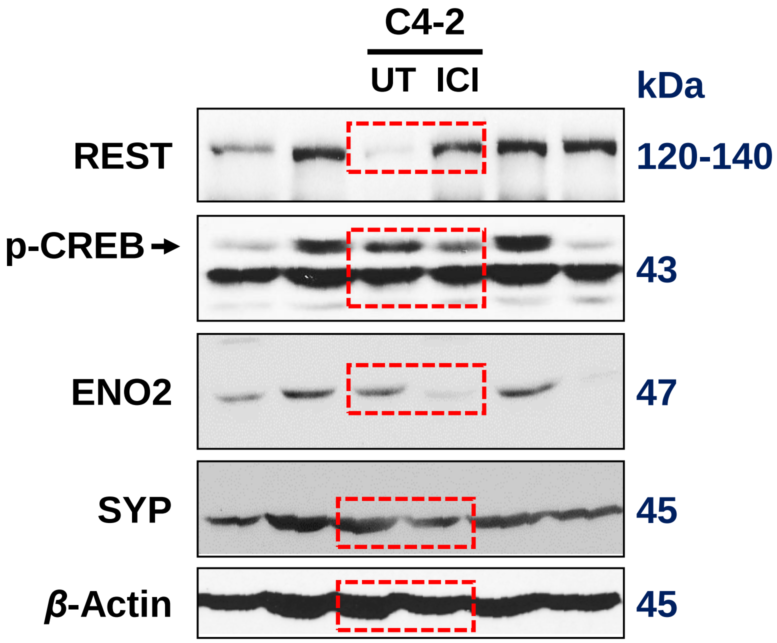

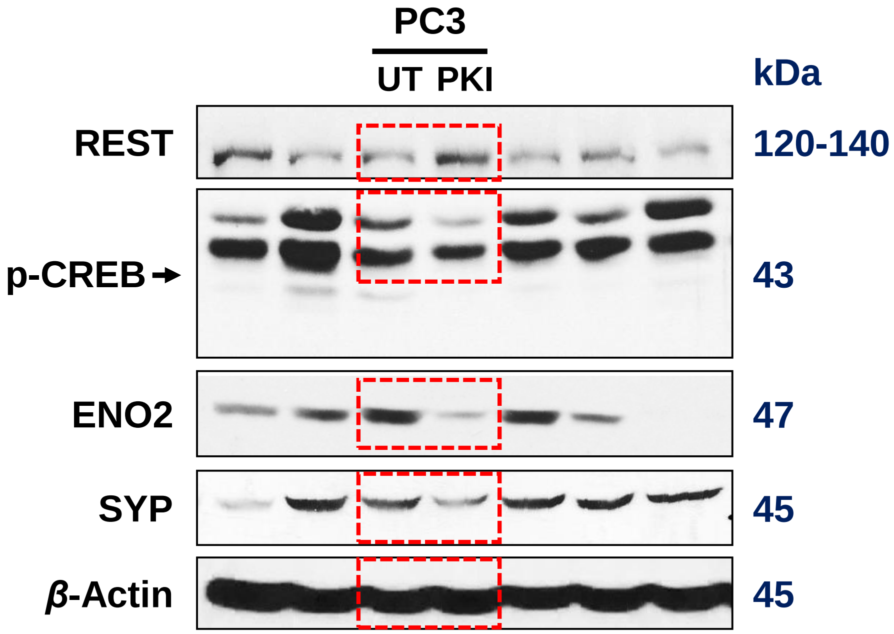

Appendix Fig. 4

Fig. 4 C

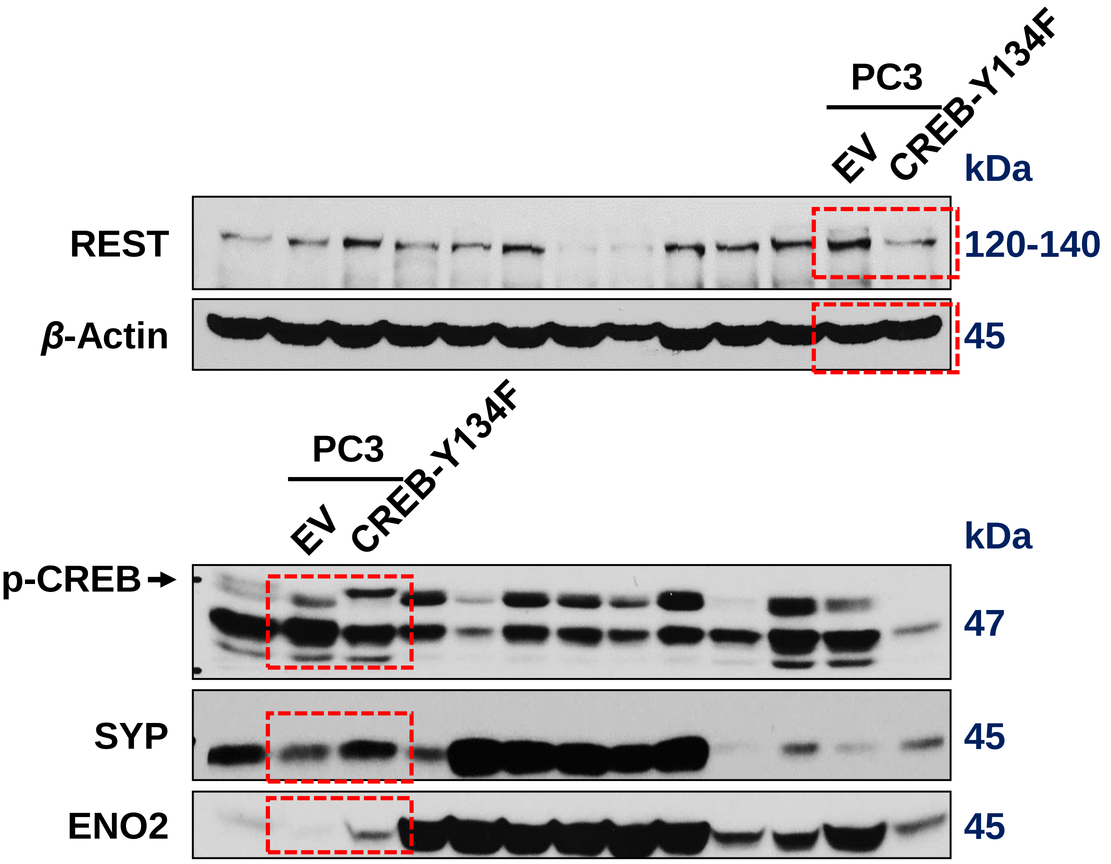

Fig. 4 D

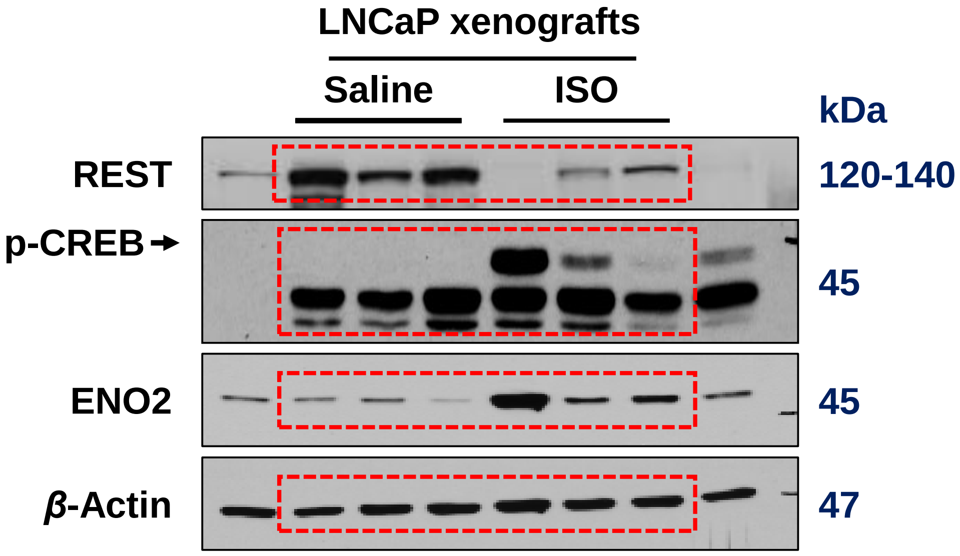

Appendix Fig. 5

Fig. 5 A

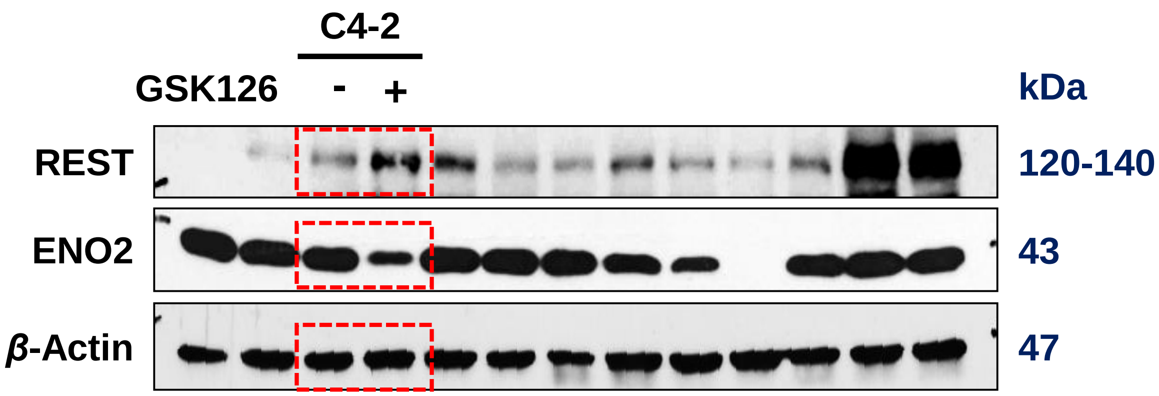

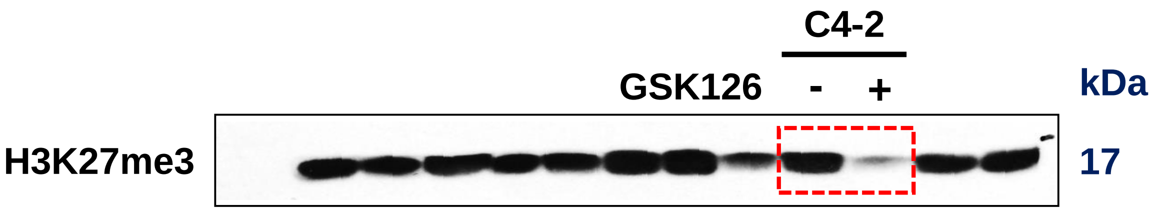

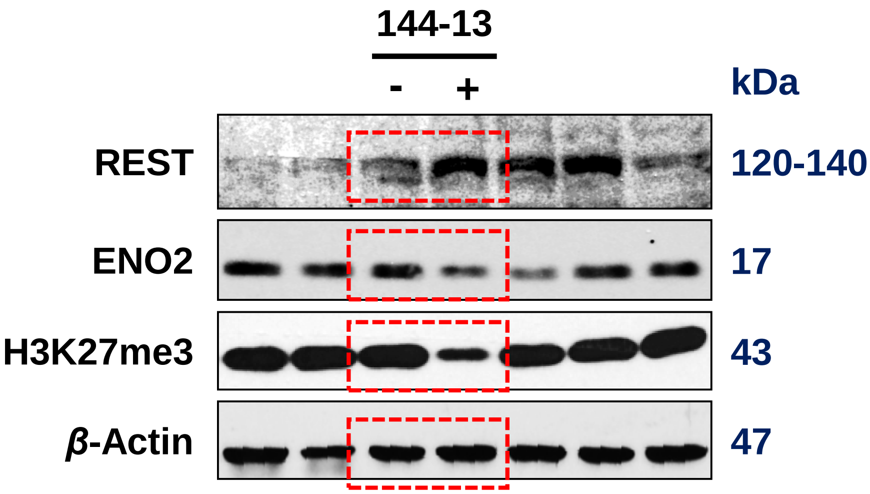

Appendix Fig. 5

Fig. 5 B

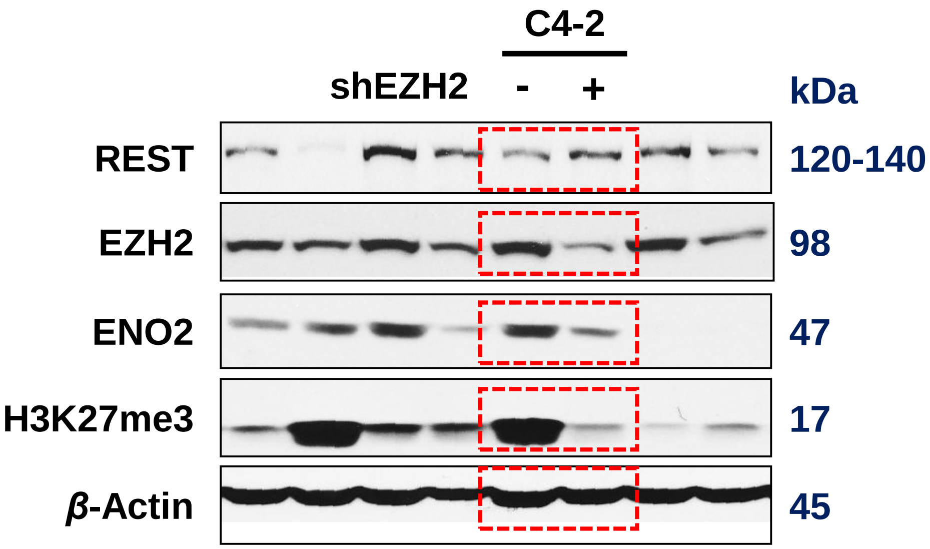

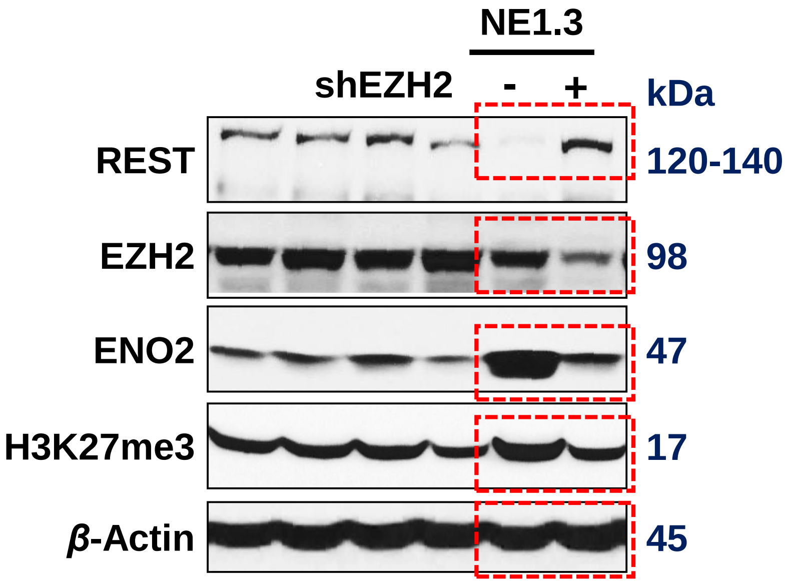

Appendix Fig. 6

Fig. 6 A

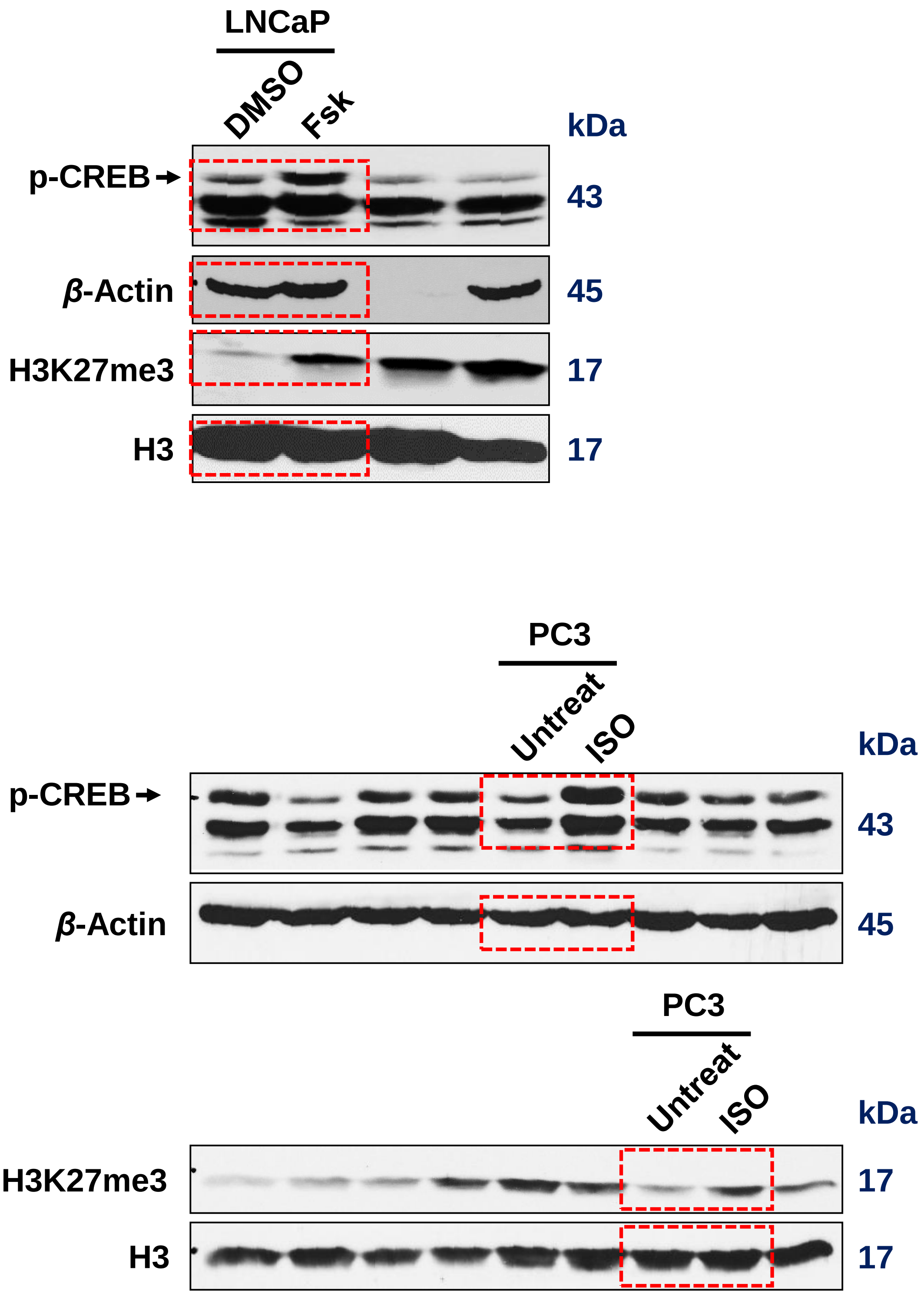

Appendix Fig. 6

Fig. 6 B

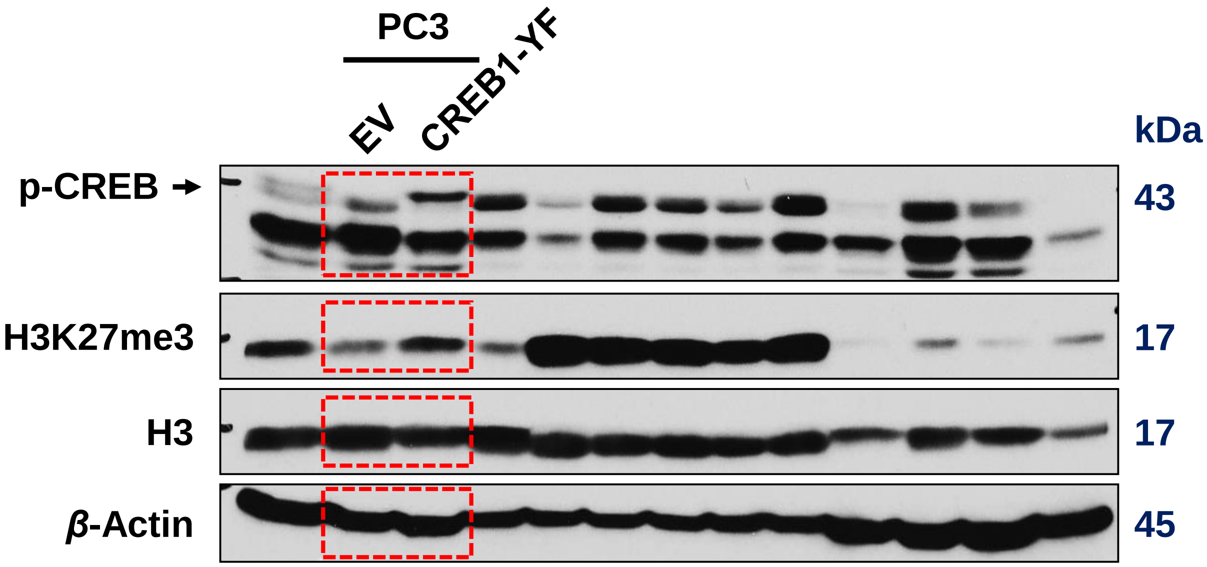

Appendix Fig. 6

Fig. 6 C

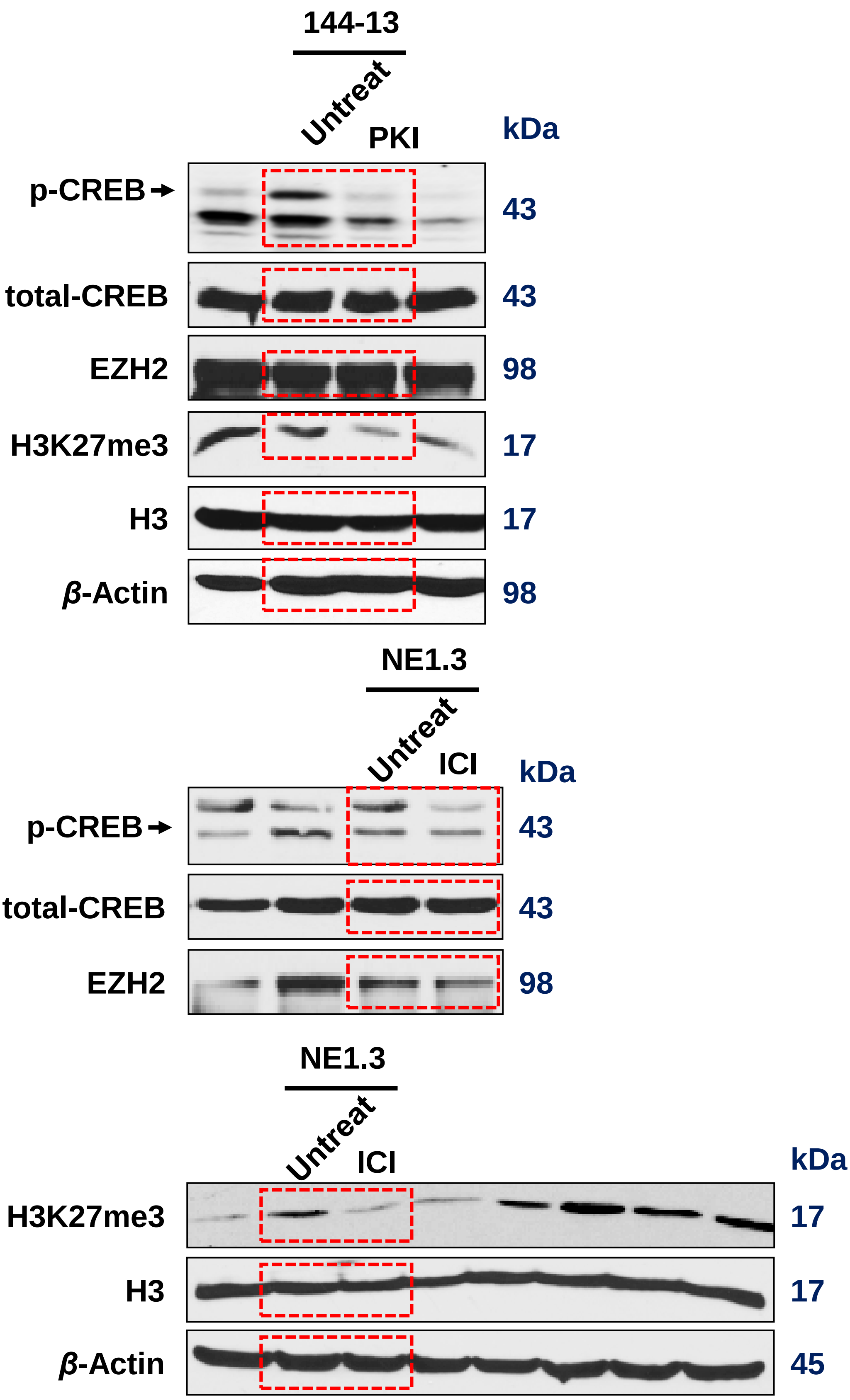

Appendix Fig. 6

Fig. 6 D

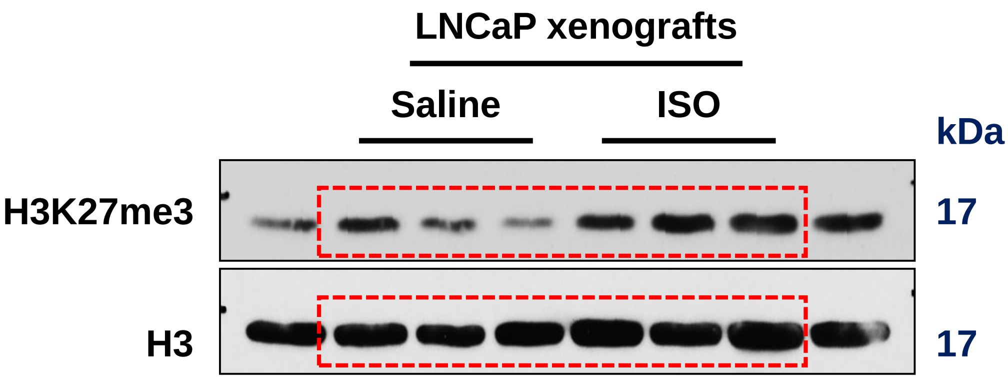

Appendix Fig. 7

Fig. 7 A

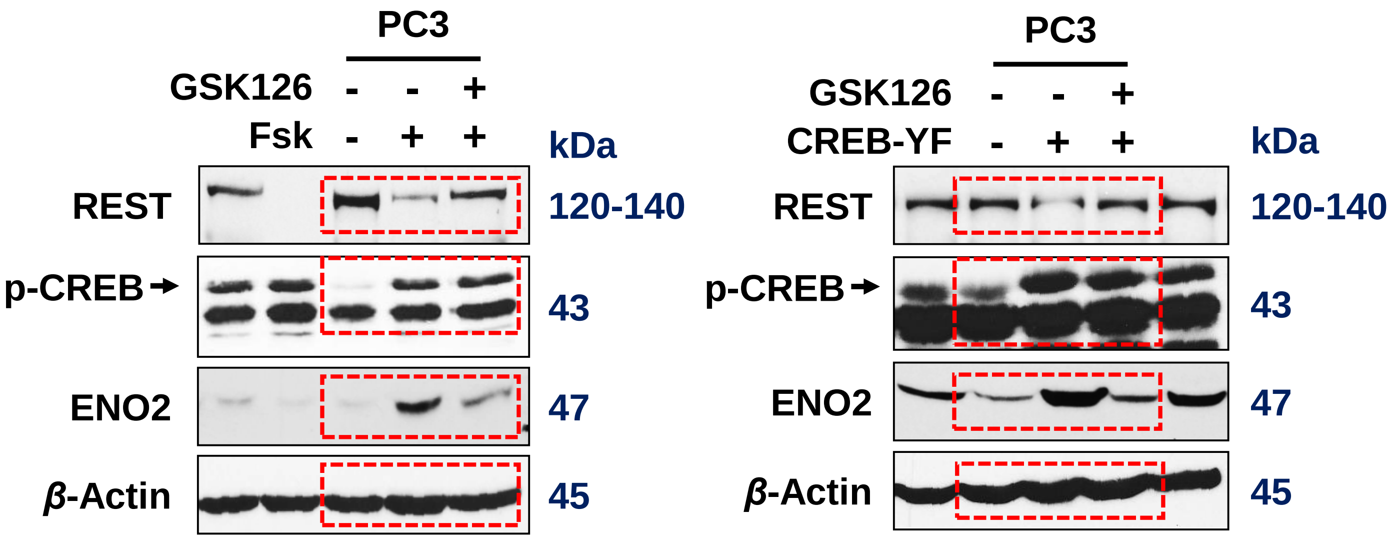

Fig. 7 B

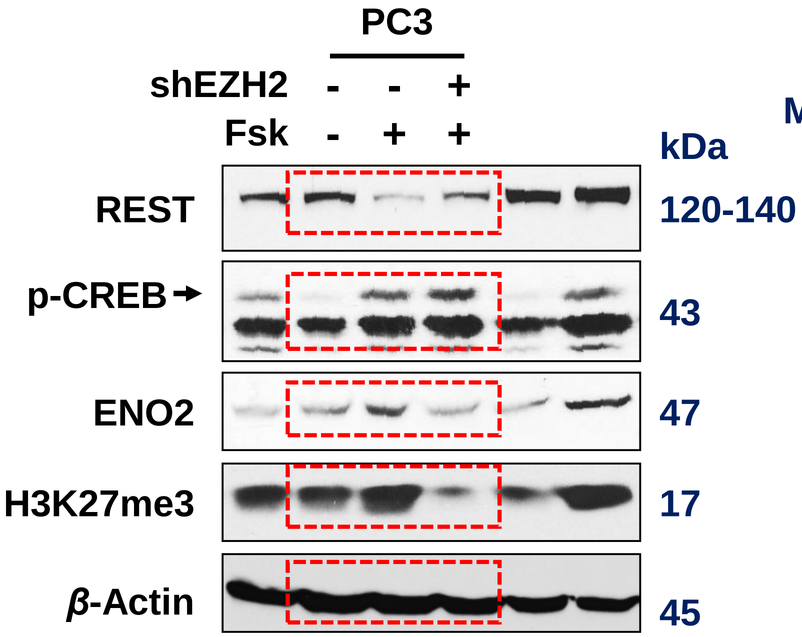

Fig. 7 D

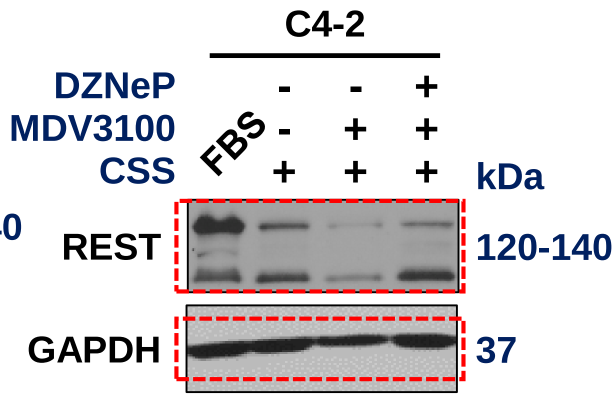

Appendix Fig. 7

Fig. 7 E

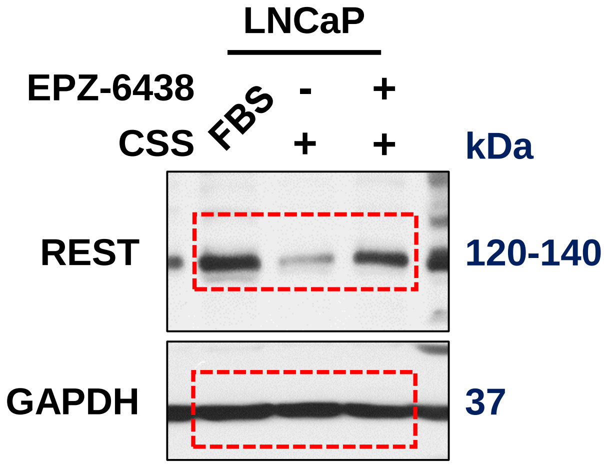

Supplement: Supplementary file 6 — Western blot -uncropped figures [file 41420_2024_2031_MOESM6_ESM.pdf]
